# The effects of inorganic nitrate and inulin co-ingestion on circulating metabolites and blood pressure in young adults: a pilot double-blind randomised crossover trial

**DOI:** 10.1017/gmb.2025.10008

**Published:** 2025-06-26

**Authors:** Jessica Virgili, Gwenaelle Le Gall, Anni Vanhatalo, Bert Bond, David Vauzour, Luciana Torquati

**Affiliations:** 1 University of Exeter Medical School, Faculty of Health and Life Sciences, Department of Public Health and Sport Sciences, University of Exeter, St Lukes Campus, Heavitree Road, Exeter EX1 2LU, UK; 2 Norwich Medical School, Faculty of Medicine and Health Sciences, University of East Anglia, Norwich NR4 7TJ, UK

**Keywords:** fermentable fibre, nitrate, gut microbiome, acetate, vascular health

## Abstract

Dietary patterns enriched in fermentable fibre (such as inulin) and inorganic nitrate are linked to cardiovascular benefits, possibly mediated by gut microbiota-derived bioactive compounds including short-chain fatty acids (SCFAs) and nitric oxide (NO). However, the potential synergistic effects remain unclear. We conducted a randomised, double-blind, crossover study to investigate the acute effects of inulin (15 g; INU), nitrate (400 mg; NO_3_^−^), and their combination (INU + NO_3_^−^) on plasma nitrate and nitrite levels, SCFAs, and blood pressure (BP) in 20 adults. Plasma nitrate and nitrite were significantly elevated following INU + NO_3_^−^ and NO_3_^−^ compared to INU (*p* < 0.001). Plasma SCFAs were increased after INU + NO_3_^−^ and INU, but the incremental AUC was not statistically significant, likely due to large inter-individual variability. No significant main effects were observed on BP; however, inverse correlations were identified between peak plasma nitrite and diastolic BP (r_s_ = −0.61, *p* = 0.004) and mean arterial pressure (MAP) (r_s_ = −0.59, *p* = 0.005) following INU + NO_3_^−^. Peak nitrate concentrations were inversely correlated with diastolic BP following NO_3_^−^ (r_s_ = −0.47, *p* = 0.004). Co-supplementation with inulin and nitrate did not enhance plasma nitrate/nitrite or BP beyond nitrate alone but modulated SCFA profiles, suggesting potential interactions between fibre fermentation and nitrate metabolism for cardiovascular health.

## Introduction

Hypertension is a significant modifiable risk factor for cardiovascular disease (CVD) and premature mortality, affecting 1.3 billion adults worldwide (Mishra et al., 2025). This highlights the crucial role of blood pressure (BP) management as a fundamental strategy for CVD prevention. Despite the availability of therapeutic interventions, approximately 80% of patients continue to have uncontrolled BP (Kario et al., 2024), underscoring the urgent need for dietary interventions (Jama et al., 2024; Norouzzadeh et al., 2025). This is particularly pertinent given the association between Western dietary patterns and the rising prevalence of hypertension (Clemente-Suárez et al., 2023). The consumption of foods rich in fermentable fibre, such as vegetables, fruits, cereal grains, and legumes, along with nitrate-rich foods such as leafy greens and beetroot, might benefit the gut microbiome and cardiovascular health (Kaye et al., 2020; Azuma et al., 2023; Wang et al., 2023; Jama et al., 2024), with subsequent health benefits likely due to the production of short-chain fatty acids (SCFAs) from fibre fractions (Boets et al., 2015) and the increased bioavailability of nitric oxide (NO) from inorganic nitrate (Norouzzadeh et al., 2025).

Inulin, a fermentable fibre mostly derived from chicory roots (Kaur et al., 2021), remains intact until it reaches the large intestine, where it undergoes fermentation into SCFAs by anaerobic bacteria, thereby promoting bacterial growth (van der Beek et al., 2018). Although direct evidence linking inulin to BP modulation is lacking, studies have suggested that inulin selectively changes gut microbiota composition (Aldubayan et al., 2023). Its consumption results in the fermentation of SCFAs within 2–12 hours, with acetate being more prevalent than butyrate or propionate (Tarini and Wolever, 2010; Boets et al., 2015; van der Beek et al., 2018). SCFAs have been associated with enhanced cardiovascular health (Xu et al., 2022), including the reversal of hypertension due to a deficiency in fermentable fibre in mice diets (Kaye et al., 2020). These microbial metabolites are absorbed from the colon into the bloodstream via monocarboxylate transporters and passive diffusion (Xu et al., 2022). In the circulation, SCFAs may reduce BP by activating G protein-coupled receptors (GPR41 and GPR43) in vascular and renal tissues, facilitating vasodilation (Xu et al., 2022). This effect has been demonstrated in preclinical studies (Kaye et al., 2020), with one human intervention study indicating that SCFA-enriched high-amylose maize starch can lower systolic blood pressure (SBP) in hypertensive patients (Jama et al., 2023). Additionally, a preclinical study demonstrated that inulin consumption ameliorated endothelial dysfunction in a hypertensive animal model by enhancing the nitric oxide synthase (NOS) pathway, improving endothelium-dependent relaxation, and increasing the phosphorylated endothelial nitric oxide synthase (eNOS) to total eNOS ratio at Ser-1177 (eNOS phosphorylation site) and NO-producing bacteria, including *E. coli* and *Bifidobacteriaceae* (Catry et al., 2018). In human umbilical vein endothelial cells (HUVECs), incubation with acetate (not derived from inulin) similarly increased NO bioavailability by stimulating eNOS phosphorylation at Ser-1177, 2–4 hours post-incubation. Phosphorylation was dependent on AMP-activated protein kinase (AMPK) activation (Sakakibara et al., 2010).

Inorganic nitrate serves as a bioactive compound that functions as a precursor to NO, a signalling molecule essential for various physiological processes (Lundberg et al., 2008). Upon ingestion, nitrate is rapidly absorbed in the upper gastrointestinal tract, with approximately 25% actively sequestered by the salivary glands (Lundberg et al., 2018). Within the oral cavity, commensal bacteria located on the tongue facilitate the reduction of nitrate to nitrite, which is subsequently swallowed and metabolised in the stomach. Under acidic gastric conditions and in the presence of specific dietary components, nitrite is chemically reduced to NO. This process, referred to as the nitrate–nitrite–NO pathway, is particularly active under hypoxic conditions (Lundberg et al., 2018). Furthermore, NO is synthesised via the oxidation of L-arginine catalysed by NOS enzymes, with eNOS being primarily responsible for NO production within the vascular system (Lundberg and Weitzberg, 2005). NO acts as a vasodilator by diffusing into the vascular smooth muscle cells and activating the soluble guanylate cyclase (sGC)/cyclic guanosine monophosphate pathway (cGMP), leading to vasodilation and a reduction in BP (Carlström et al., 2018). Consumption of dietary nitrate, either as a salt, green leafy vegetables, or through beetroot juice supplementation, has been consistently shown to lower BP within a timeframe ranging from a few hours to several weeks (Siervo et al., 2013; Ashworth and Bescos, 2017; Bahadoran et al., 2017). This strategy is proposed as a cost-effective means of preventing cardiovascular disease, particularly in adults with elevated baseline BP or chronic conditions such as hypertension (Ashworth and Bescos, 2017). However, this perspective has recently been questioned, with suggestions that other factors, such as oral health, might play a more crucial role in BP regulation, and that the only significant connection appears to be between salivary nitrate and BP (Bescos et al., 2025).

Although plasma nitrate and nitrite are well-established precursors for NO production and bioavailability (Kapil et al., 2010), the influence of inulin on NO bioavailability via eNOS, as evidenced in *in vitro* and animal studies (Sakakibara et al., 2010; Catry et al., 2018) or described hypothetically through modulation of the gut microbiota, potentially enhancing NO bioavailability (González-Soltero et al., 2020), remains uncertain. Metabolites derived from inulin and nitrate have the potential to lower BP via distinct biological mechanisms, as previously described. However, whether their combined intake exhibits complementary or synergistic effects remains unknown, as evidence from human studies is currently lacking. Consequently, investigating the response of their respective metabolites to co-supplementation could inform future research aimed at enhancing vascular health in at-risk populations, including adults with hypertension.

The primary aim of this study was to explore whether the combination of nitrate and inulin affects plasma nitrate and nitrite compared to the effects of consuming these supplements individually. The secondary aim was to assess the independent and combined effects of these supplements on the production of SCFAs. Furthermore, this study sought to examine the potential impact on BP when peak concentrations of nitrate, nitrite, and acetate were reached, following nitrate and inulin supplementation separately or together. We hypothesised that combining inulin and nitrate would lead to higher plasma nitrite than when each supplement was consumed alone and that peak plasma nitrite concentrations would be inversely correlated with BP, resulting in a significant reduction in BP.

## Methods

### Study design

This study was a double-blind, randomised, crossover design. Participants were initially screened via a video call, and those who expressed interest and provided written informed consent underwent a laboratory-based screening procedure. This procedure included anthropometric measurements (height and body weight), a comprehensive medical history report, and office BP measurements. The primary inclusion criteria were adults aged between 18 and 45 years, with a body mass index (BMI) ranging from 18 to 25 kg/m^2^ and BP measurements within the normotensive range, defined as a SBP of ≤120 mmHg and a diastolic blood pressure (DBP) of ≤80 mmHg (McEvoy et al., 2024). Participants were excluded if they had been taking antibiotics for three months prior to or during the study, were engaged in a weight loss intervention or adhered to any restrictive dietary practices (e.g., vegan, FODMAP, etc.), had a history of chronic gastrointestinal conditions, or pre-existing medical conditions, including hypertension, diabetes, cardiovascular or dental conditions requiring treatment. Additional exclusion criteria included regular use of antibacterial mouthwash or tongue scrapes, smoking, and consumption of prebiotics, probiotics, or nitrate supplements for at least one month prior to or during the study (see Supplementary Methods for a comprehensive list of exclusion criteria). The study protocol adhered to the core principles of the ICH-GCP and the Helsinki Declaration and was approved by the Public Health and Sport Sciences Ethics Committee (University of Exeter; approval number: 22–02-02 A-01).

### Study procedures

All tests were performed at the University of Exeter, Faculty of Health and Life Sciences. Twenty normotensive participants attended three separate visits where they were randomly assigned to receive either 15 g Orafti inulin (Orafti Chicory Inulin powder, Hellenia, UK) (INU), 400 mg potassium nitrate (NO_3_^−^) (Vital Minerals, UK) (NO_3_^−^ condition), or a combination of both (INU + NO_3_^−^) in a randomised order. The nutritional composition of the conditions is shown in Supplementary Table S1. The doses of 15 g inulin and 400 mg nitrate were based on previous studies (Boets et al., 2015; Kapil et al., 2015). For inulin, we selected a dose previously demonstrated to be well tolerated regarding reduced risk of gastrointestinal adverse effects, including abdominal distension, nausea, flatulence, constipation, and gastrointestinal cramping and rumbling (Bonnema et al., 2010). The 400 mg nitrate dose represents a practical amount consumable through vegetables (Li et al., 2024) and is within the range shown to be well tolerated and offer vascular benefits (Kapil et al., 2015). A seven-day washout period between visits ensured nitrate and nitrite levels returned to pre-supplementation levels (Capper et al., 2022) and eliminated potential carry-over effects from inulin supplementation (Depeint et al., 2008).

**Figure 1.**
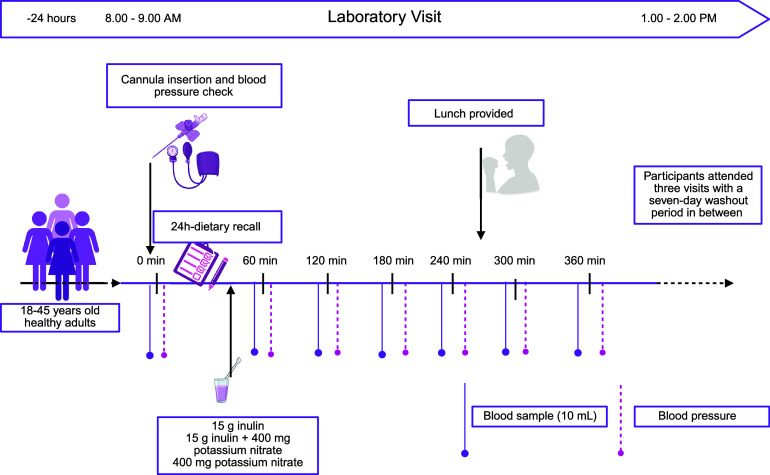
Study visit overview, including timings of blood sampling and blood pressure measurements. Created with BioRender.com.

Visits occurred between 08:00 AM and 2:00 PM after a 12-hour overnight fast (water permitted). Participants received a list of nitrate-rich foods to avoid the day before and were instructed to avoid caffeine for 12 hours, vigorous exercise, and alcohol within 24 hours of the visit. Compliance was verified through a 24-hour dietary recall analysed using Nutritics software (Nutritics, 2019, Research Edition v6.04). At each visit, a cannula was inserted into an antecubital vein for baseline blood collection, followed by BP measurement. Participants consumed the allocated supplement(s) within 5 minutes. Blood samples were collected at 60, 120, and 180 minutes post-consumption, followed by a low-fibre, low-nitrate meal within 10 minutes (94 g white bread, 22 g lactose-free cheddar cheese, and 7 g spreadable plant-based butter). The nutritional composition of the standard meal is presented in Supplementary Table S2. Additional blood samples were obtained at 240, 300, and 360 minutes with BP measurements (Figure 1).

### Blood pressure measurements and anthropometric measurements

Clinic BP measurements were taken according to previously described guidelines (Muntner et al., 2019). BP was measured four times with a one-minute rest between readings using an electronic sphygmomanometer (Dinamap Pro; GE Medical System, Tampa, FL) and appropriately sized upper-arm cuff after a 10-minute rest period. The readings were blinded to the participant, and an average of the last three measurements at each timepoint was taken for analysis. Participants’ body mass and height were measured using a digital balance scale (precision of 0.1 kg) and a wall-mounted stadiometer (accurate to 0.1 cm). During these measurements, the participants were asked to remove their shoes and wear minimal clothing.

### Blood sampling

Whole blood samples were obtained through a cannula and collected in 10 mL lithium heparin vacutainers (BD). Following each blood sample collection, 5 mL of non-coagulant saline solution was injected through the cannula to prevent clotting and line blockage. The samples were centrifuged immediately at 3,300 × g for 10 min at 4 °C, and the plasma was divided into aliquots and frozen at −80 °C. Plasma samples were used to measure concentrations of nitrate, nitrite, and SCFAs.

### Plasma nitrate and nitrite analysis

Plasma nitrate and nitrite samples were deproteinised by cold ethanol precipitation prior to analysis. Briefly, plasma samples were immediately frozen at −80 °C for the subsequent determination of nitrate and nitrite. Each sample was mixed with cold ethanol at a ratio of 1:2 (sample: ethanol) and centrifuged at 13,000 rpm (4 °C) for 15 minutes to precipitate proteins. The supernatant was then analysed for nitrate and nitrite concentrations using a Sievers gas-phase chemiluminescence nitric oxide analyser (NOA 280i, Analytix), in accordance with a previously outlined methodology (Piknova et al., 2016), as previously described (Wylie et al., 2013).

### Plasma short-chain fatty acids analysis

Plasma SCFAs levels were measured using LC–MS/MS, as previously described (Dei Cas et al., 2020). Briefly, 40 μL of plasma was diluted with 500 μL of ice-cold methanol, followed by incubation on dry ice and centrifugation at 14,800 rpm for 5 minutes. The supernatants were filtered, and the extracts were evaporated using Savant™ SpeedVac™, followed by reconstitution with 40 μL of methanol. To each 20 μL of the reconstituted sample, an internal standard mix (acetic acid d3, propionic acid d2, and isobutoxyacetic acid) was added. For derivatisation, 10 μL of 3-NPH and 10 μL of EDC were added, and the mixture was incubated at 37 °C for 30 minutes, followed by quenching with 20 μL of 0.1% formic acid. The derivatised samples were then transferred to autosampler vials and subjected to LC–MS/MS analysis. Stock solutions of the metabolites in methanol were prepared and stored at −80 °C. Calibration standards, including acetic acid, propionic acid, and other SCFAs, were run at the beginning, middle, and end of each analytical queue to construct calibration curves based on the analyte–internal standard response ratios, facilitating the accurate quantification of SCFAs. Detailed information on the procedure for analysing SCFAs in plasma samples can be found in the Supplementary Methods section.

### Sample size

This pilot study sought to offer initial insights into the feasibility of these interventions and their overall impact on both the primary and secondary outcomes. The study sample size was determined on the basis of two key considerations. Firstly, previous studies have demonstrated positive effects of various types and doses of inorganic nitrate supplementation on plasma nitrite levels in healthy adults (McDonagh et al., 2018; Jakubcik et al., 2021). Second, we used the predicted effect size estimates proposed by Whitehead et al. (2016) to calculate the sample size for a pilot trial. According to these recommendations, a sample size of 20 participants per group would be sufficient to detect a small effect size (δ = 0.10–0.30) with a power of 0.80 and a p-value of less than 0.05.

### Statistical analysis

Data were analysed using SPSS (IBM SPSS Statistics, Version 29) and visualised using GraphPad Prism (GraphPad Software V 10.1.1; San Diego, CA, USA). Two-way repeated measures ANOVA assessed time × treatment effects on plasma nitrite, nitrate, and SCFAs, with Bonferroni correction for timepoint comparisons. For non-significant interactions, main effects of time and condition were analysed separately. Total and incremental areas under the curve (tAUC and iAUC) were calculated using the trapezium rule via R Statistical Software, with differences analysed using one-way repeated-measures ANOVA and Bonferroni post-hoc test. For non-normal data with violated sphericity, the Friedman test was used (Blanca et al., 2023). Spearman’s and Pearson’s correlations examined relationships between nitrite, nitrate, acetate, and BP variables, with strengths categorised as weak (0.2), moderate (0.5), and strong (0.8) (Mukaka, 2012). Data are expressed as mean ± SD, with significance at p ≤ 0.05. For full statistical methods, see online supplementary methods.

## Results

### Participants characteristics

**Figure 2.**
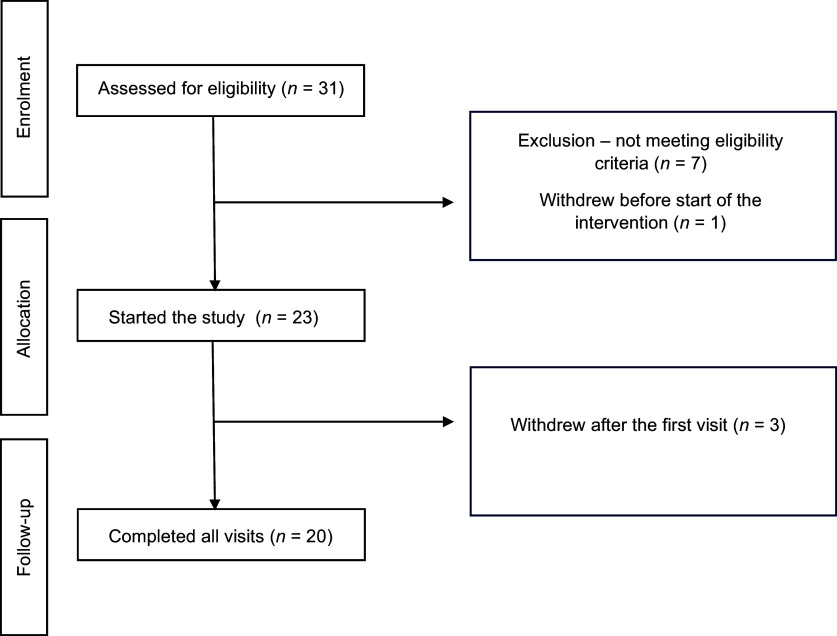
CONSORT diagram flowchart for the recruitment and retention of the study participants.

Figure 2 presents a flowchart of participants’ recruitment, and Table 1 presents their baseline characteristics. Twenty normotensive participants with an average age of 27.4 ± 6.3 years (mean ± SD), a BMI of 24.6 ± 3.1 kg/m^2^, and a waist circumference of 81.5 ± 8.5 cm were included in the study. Three participants discontinued the intervention due to time constraints, which prevented the completion of the remaining two visits. None of the participants reported any adverse reactions or discomfort after consuming supplements during the study visits. All participants adhered to a low-nitrate diet according to their completed food diaries. No significant differences were observed in micronutrient, dietary fibre, or macronutrient intake across the three laboratory visits, except for the percentage of fat intake for the total daily energy consumption (Table 2).

**Table 1.** Baseline values of the participants (*n* = 20)

Characteristics (*n* = 20)	
Age (y)	27.4 ± 6.3
Male:Female	11:9
Height (cm)	172.4 ± 9.8
Body weight (kg)	73.5 ± 12.5
BMI (kg/m^2^)	24.6 ± 3.1
Waist (cm)	81.5 ± 8.5
Waist-to-hip ratio	0.8 ± 0.1
Resting SBP (mmHg)	114.4 ± 8.8
Resting DBP (mmHg)	62.7 ± 6.2
Resting MAP (mmHg)	82.3 ± 6.5
Plasma NO_2_^−^ (nM)	72.3 ± 29.3
Plasma NO_3_^−^ (μM)	24.4 ± 9.3
Plasma acetate (μM)	79.3 ± 19.3
Plasma butyrate (μM)	0.6 ± 0.3
Plasma propionate (μM)	2.2 ± 0.4
Ethnic group	18 Caucasian, 2 South Asian

Data are expressed as means ± standard deviation. BMI, body mass index; SBP, systolic blood pressure, DBP, diastolic blood pressure; MAP, mean arterial pressure; ethnic group, self-declared.

**Table 2.** Macronutrient, micronutrient, and fibre intake data from the 24-h dietary recalls from 20 normotensive young adults participating in the study before each laboratory visit

Nutrient	INU + NO_3_^−^	NO_3_^−^	INU	*p*-value
Energy (kcal/day)	1841.3 ± 473.4	1784.7 ± 471.5	1878.0 ± 665.0	0.545
Carbohydrates (%E kcal)	46.0 ± 9.8	43.5 ± 9.6	43.6 ± 9.8	0.274
Carbohydrate (g/day)	210.2 ± 63.2	193.1 ± 63.8	199.2 ± 67.7	0.300
Protein (%E kcal)	17.3 (8.8)	17.1 (8.7)	16.8 (6.8)	0.253
Protein (g/day)	89.6 ± 29.2	83.6 ± 31.1	82.8 ± 35.3	0.360
Fat (%E kcal)	34.0 ± 7.6	37.2 ± 8.1	38.5 ± 8.6	0.021*
Fat (g/day)	71.4 ± 29.9	75.3 ± 28.2	83.3 ± 47.3	0.194
Saturated fat (g/day)	22.9 (12.9)	27.1 (16.3)	24.8 (23.3)	0.287
Cholesterol (mg/day)	313. 1 ± 262.4	326.5 ± 353.1	237.7 ± 319.4	0.468
Dietary fibre (g/day)	18.2 ± 6.9	17.3 ± 6.7	18.2 ± 8.9	0.756
Potassium (mg/day)	1414.7 ± 964.6	1397.0 ± 631.7	1572.9 ± 919.2	0.522
Magnesium (mg/day)	200.6 ± 103.9	191.9 ± 65.9	195.9 ± 100.7	0.896
Vitamin E (mg/day)	4.8 ± 4.8	4.0 ± 2.5	5.1 ± 4.1	0.286
Vitamin C (mg/day)	10.5 ± 15.6	12.7 ± 15.5	17.3 ± 26.1	0.493

Data are presented as mean ± SD or median (IQR) for non-normally distributed variables. Energy adjusted values (%E kcal) are expressed as the percentage of total energy contributed by the nutrient. * *P* value <0.05 indicate a significant difference between the INU + NO_3_^−^ and the INU conditions.

### Plasma nitrate and nitrite

**Figure 3.**
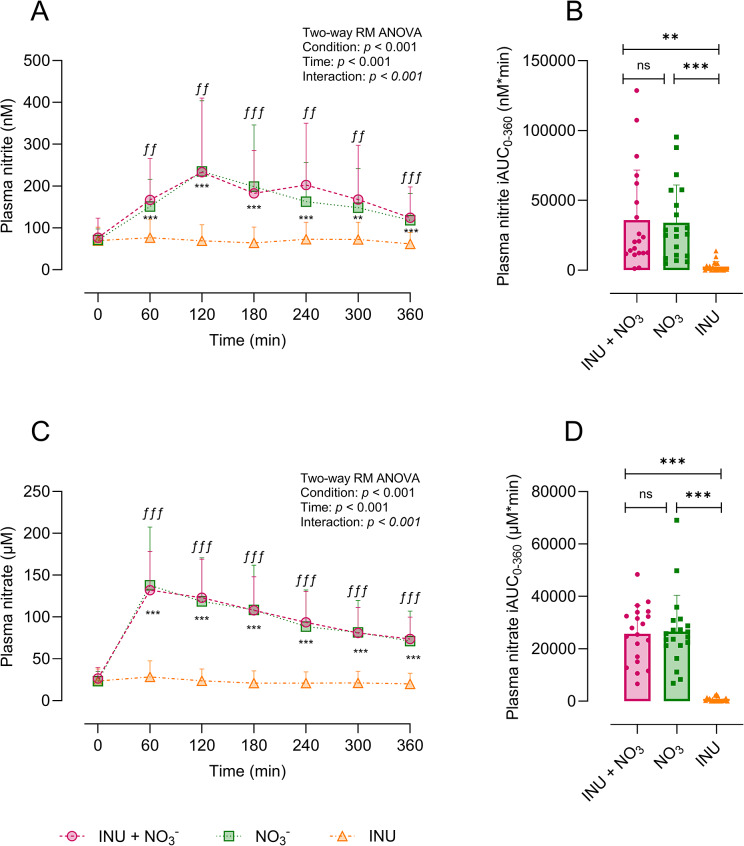
Plasma nitrite and nitrate following INU + NO_3_^−^ (pink), NO_3_^−^ (green), and INU (orange) conditions. (A) Plasma nitrite (nM) over 360 minutes; (B) plasma nitrite iAUC from baseline to 360 minutes; (C) plasma nitrate (μM) over 360 minutes; (D) plasma nitrate iAUC from baseline to 360 minutes. All results are expressed as means ± SD (*n* = 20). ^ƒ^Significant differences between INU + NO_3_^−^ and INU. *Significant differences between NO_3_^−^ and INU. ns *p* > 0.05, **p = 0.001, ***p < 0.001. Abbreviations: iAUC, incremental area under the curve; INU, inulin; NO_3_^−^, nitrate; min, minutes.

Plasma nitrite and nitrate did not differ between the baseline conditions (*p* > 0.05). The rise in plasma nitrite above baseline following both INU + NO_3_^−^ and NO_3_^−^ reached peak concentrations at 120 minutes (233 ± 177 nM, 95% CI, 150–315 nM and 235 ± 169 nM, 95% CI, 156–313 nM, respectively), in contrast to INU (69 ± 38 nM, 95% CI, 50–87 nM) (*p* = 0.001 and *p* < 0.001, respectively) (Figure 3A). The iAUC for plasma nitrite was 35883 ± 35914 nM*min (95% CI, 19074–52691 nM*min) and 33990 ± 27030 nM*min (95% CI, 21339–46640 nM*min) following INU + NO_3_^−^ and NO_3_^−^, respectively, both significantly exceeding the nitrate iAUC following INU (2520 ± 3685 nM*min, 95% CI, 795–4244 nM*min, *p* < 0.001) (Figure 3B). A similar trend was observed for nitrite tAUC across all three conditions (Supplementary Figure S1A). The increase in plasma nitrate above baseline following both INU + NO_3_^−^ and NO_3_^−^ reached peak concentrations at 60 minutes (132 ± 45 μM, 95% CI, 110–153 μM and 137 ± 69 μM, 95% CI, 105–170 μM, respectively), compared to INU (28 ± 19 μM, 95% CI, 19–37 μM) (*p* < 0.001 for both) (Figure 3C). Nitrate iAUC after INU+ NO_3_^−^ (25709 ± 10849 μM*min, 95% CI, 20631–30787 μM*min) was comparable to that of NO_3_^−^ (26584 ± 13843 μM*min, 95% CI, 20105–33063 μM*min), both significantly greater than the nitrate iAUC after INU (719 ± 829 μM*min, 95% CI, 331–1107 μM*min, *p* < 0.001) (Figure 3D). A similar pattern was observed for nitrate tAUC across all three conditions (Supplementary Figure S1B).

### Plasma short-chain fatty acids

**Figure 4.**
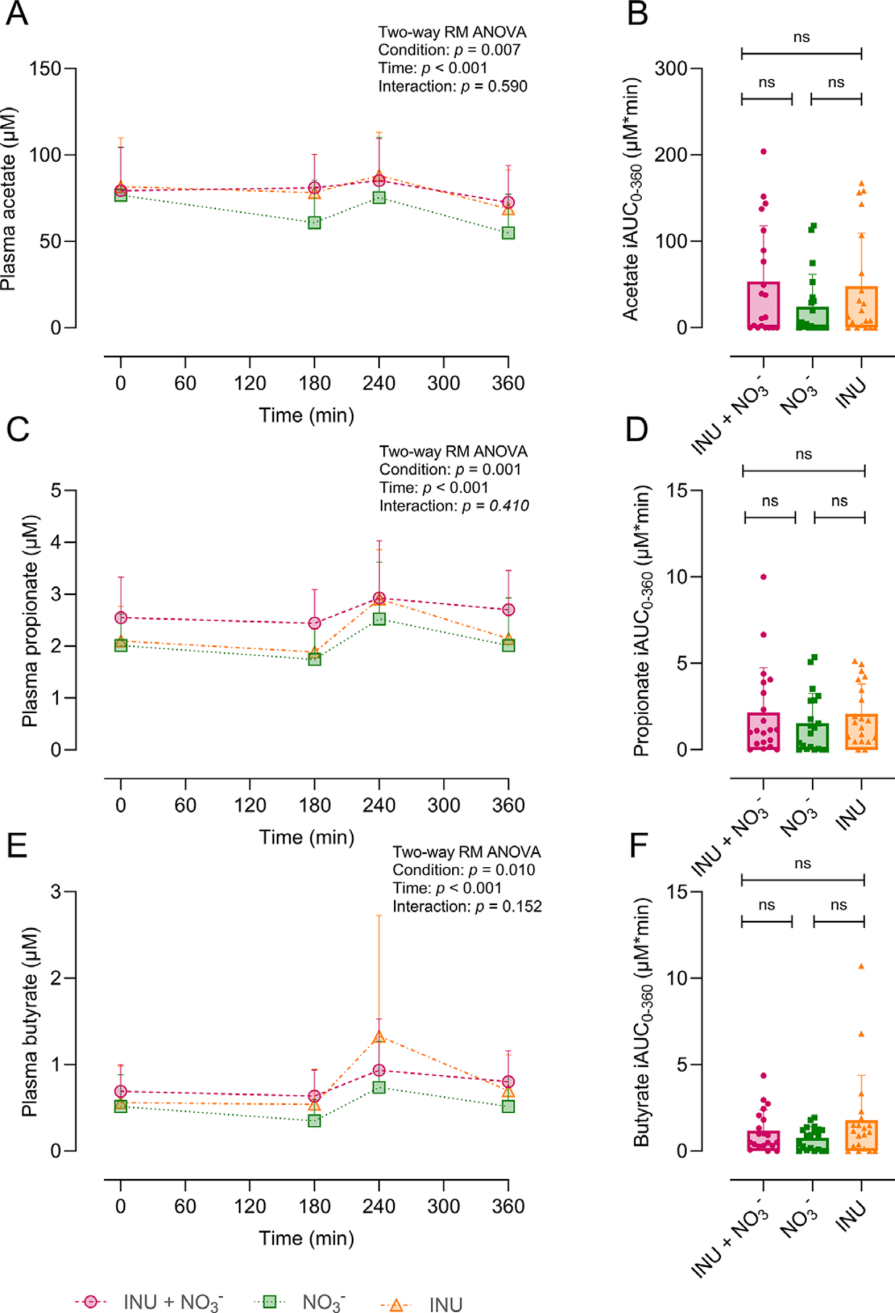
Plasma acetate, propionate, and butyrate following INU + NO_3_^−^ (pink), NO_3_^−^ (green), and INU (orange) supplements. (A) Plasma acetate (μM) over 360 minutes; (B) plasma acetate iAUC from baseline to 360 minutes; (C) plasma propionate (μM) over 360 minutes; (D) plasma propionate iAUC from baseline to 360 minutes; (E) plasma butyrate (μM) over 360 minutes; (F) plasma butyrate iAUC from baseline to 360 minutes. All results are expressed as means ± SD (*n* = 20). ns *p* > 0.05. Abbreviations: iAUC, incremental area under the curve; INU, inulin; NO_3_^−^, nitrate; min, minutes.

Plasma SCFAs concentrations did not differ between the baseline conditions (*p* > 0.05). The mean plasma acetate was 79.56 ± 13.06 μM (95% CI: 73.45–85.67 μM) for INU + NO_3_^−^, 79.23 ± 17.48 μM (95% CI: 71.05–87.41 μM) for INU, and 66.97 ± 21.42 μM (95% CI: 56.94–77.00 μM) for NO_3_^−^. Plasma acetate was significantly higher in the INU + NO_3_^−^ (mean difference: 12.59 ± 18.60 μM, 95% CI: 1.66–23.52 μM, *p* = 0.021) and INU (mean difference: 12.26 ± 20.48 μM, 95% CI: 0.23–24.29 μM, *p* = 0.045) compared to the NO_3_^−^ (Figure 4A). Plasma acetate iAUC was higher following INU + NO_3_^−^ (53.47 ± 64.57 μM, 95% CI, 23.25–83.69 μM) and INU (47.86 ± 61.66 μM, 95% CI, 19.00–76.72 μM) compared to NO_3_^−^ (24.38 ± 37.58 μM, 95% CI, 6.80–41.96 μM); however, these differences did not reach statistical significance (*p* = 0.241) (Figure 4B). In contrast, plasma tAUC was significantly higher following INU + NO_3_^−^ and INU compared to NO_3_^−^ (*p* = 0.020 and *p* = 0.037, respectively) (Supplementary Figure S2A). The mean plasma propionate was 2.73 ± 0.63 μM (95% CI: 2.45–3.01 μM) for INU + NO_3_^−^, 2.26 ± 0.45 μM (95% CI: 2.04–2.47 μM) for INU, and 2.07 ± 0.63 μM (95% CI: 1.79–2.36 μM) for NO_3_^−^. Plasma propionate concentrations were significantly higher following INU + NO_3_^−^ compared to NO_3_^−^ (mean difference: 0.66 ± 0.89 μM, 95% CI: 0.15–0.17 μM, *p* = 0.010), while INU and NO_3_^−^ showed no significant difference (mean difference: 0.18 ± 0.54 μM, 95% CI: −0.13-0.50 μM, *p* = 0.416) (Figure 4C). Plasma propionate iAUC was higher following INU + NO_3_^−^ (2.16 ± 2.59 μM, 95% CI, 0.95–3.38 μM) and INU (2.08 ± 1.74 μM, 95% CI, 1.26–2.89 μM) compared to NO_3_^−^ (1.53 ± 1.72 μM, 95% CI, 0.72–2.34 μM), although this difference was not statistically significant (*p* = 0.591) (Figure 4D). Plasma propionate tAUC was significantly higher following INU + NO_3_^−^, but not INU, compared to NO_3_^−^ (*p* = 0.010 and *p* = 0.379, respectively) (Supplementary Figure S2B). The mean plasma butyrate concentrations were 0.81 ± 0.31 μM (95% CI: 0.66–0.96 μM) for INU + NO_3_^−^, 0.76 ± 0.49 μM (95% CI: 0.52–0.99 μM) for INU, and 0.50 ± 0.22 μM (95% CI: 0.39–0.60 μM) for NO_3_^−^. Plasma butyrate concentrations were significantly higher following INU + NO_3_^−^ (mean difference: 0.32 ± 0.22 μM, 95% CI: 0.18–0.46 μM, *p* < 0.001) and tended to be higher following INU (mean difference: 0.26 ± 0.45 μM, 95% CI: −0.01-0.53 μM, *p* = 0.058) compared to the NO_3_^−^ condition (Figure 4E). Plasma butyrate iAUC was higher following INU + NO_3_^−^ (1.18 ± 1.19 μM, 95% CI, 0.62–1.74 μM) and INU (1.79 ± 2.61 μM, 95% CI, 0.56–3.01 μM), compared to NO_3_^−^ (0.76 ± 0.65 μM, 95% CI, 0.45–1.06 μM), although this difference was not statistically significant (*p* = 0.166) (Figure 4F). Plasma butyrate tAUC was significantly higher following INU + NO_3_^−^ and INU compared to NO_3_^−^ (*p* = 0.008) (Supplementary Figure S2C).

### Resting blood pressure

There were no significant baseline differences in SBP, DBP, and mean arterial pressure (MAP) across the various conditions (*p* > 0.05) (Table 3). SBP remained stable over time and did not differ between conditions. However, DBP and MAP exhibited significant changes over time (*p* < 0.001 and *p* = 0.022, respectively), without any effects from the conditions or interactions. No associations were identified between acetate and BP variables after INU or INU + NO_3_^−^. When NO_3_^−^ was consumed alone, peak plasma nitrite did not correlate with BP. In contrast, following INU + NO_3_^−^, peak plasma nitrite showed a moderate negative correlation with DBP (r_s_ = −0.61, *p* = 0.004) and MAP (r_s_ = −0.59, *p* = 0.005). Additionally, a moderate negative correlation was found between DBP and plasma nitrate following NO_3_^−^ (r_s_ = −0.47, *p* = 0.04) (Table 4).

**Table 3.** Mean ± SD of office blood pressure measurements following consumption of the three supplements in normotensive adults (*n* = 20)

Timepoint	SBP INU + NO_3_^−^	SBP NO_3_^−^	SBP INU	DBP INU + NO_3_^−^	DBP NO_3_^−^	DBP INU	MAP INU + NO_3_^−^	MAP NO_3_^−^	MAP INU
Baseline (mmHg)	113 ± 11	116 ± 9	114 ± 10	62 ± 6	63 ± 6	64 ± 7	81 ± 8	83 ± 6	82 ± 8
60 min (mmHg)	115 ± 11	116 ± 7	115 ± 11	64 ± 6	66 ± 7	63 ± 7	83 ± 7	84 ± 6	82 ± 8
120 min (mmHg)	115 ± 10	114 ± 8	114 ± 10	63 ± 6	64 ± 6	62 ± 6	82 ± 7	82 ± 6	82 ± 7
180 min (mmHg)	117 ± 9	116 ± 10	117 ± 9	64 ± 7	64 ± 6	64 ± 7	84 ± 6	83 ± 7	83 ± 7
240 min (mmHg)	115 ± 9	114 ± 7	116 ± 8	61 ± 5	61 ± 5	61 ± 5	81 ± 5	81 ± 5	82 ± 5
300 min (mmHg)	113 ± 8	113 ± 7	116 ± 9	62 ± 5	61 ± 5	61 ± 5	81 ± 5	80 ± 5	82 ± 6
360 min (mmHg)	113 ± 8	116 ± 8	114 ± 8	62 ± 4	62 ± 5	62 ± 6	81 ± 5	82 ± 5	81 ± 6
*p*-value (time)	0.374			< 0.001***		0.022*			
*p*-value (condition)	0.794			0.903		0.795			
*p*-value (interaction)	0.394			0.401		0.568			

*p < 0.05,**p < 0.01. Abbreviations: SBP, systolic blood pressure; DBP, diastolic blood pressure; MAP, mean arterial pressure; INU, inulin, NO_3_^−^, nitrate; min, minutes.

**Table 4.** Correlation coefficients of peak changes in plasma nitrite, nitrate, and acetate with their corresponding blood pressure variables following acute ingestion of the three supplements

Metabolites: treatment	ΔSBP	ΔDBP	ΔMAP
Δ Peak plasma nitrite: INU + NO_3_^−^	r_s_ 0.02, *p* = 0.955	**r**_**s**_ − 0.61, *p* = 0.004**	**r**_**s**_ − 0.59, *p* = 0.005**
Δ Peak plasma nitrite: NO_3_^−^	r_s_ − 0.41, *p* = 0.06	−0.13, *p* = 0.56	−0.29, *p* = 0.20
Δ Peak plasma nitrate: INU + NO_3_^−^	r_s_ 0.29, *p* = 0.22	r_s_ 0.13, *p* = 0.58	r_s_ − 0.02, *p* = 0.94
Δ Peak plasma nitrate: NO_3_^−^	r_s_ 0.24, *p* = 0.32	**r**_**s**_ − 0.47, *p* = 0.04*	r_s_ − 0.06, *p* = 0.79
Δ Peak plasma acetate: INU + NO_3_^−^	r 0.20, *p* = 0.39	r 0.13, *p* = 0.57	r 0.20, *p* = 0.41
Δ Peak plasma acetate: INU	r − 0.21, *p* = 0.37	r − 0.33, *p* = 0.15	r − 0.30, *p* = 0.20

Δ changes in systolic blood pressure (ΔSBP), diastolic blood pressure (ΔDBP), and mean arterial pressure (ΔMAP). Δ changes in peak nitrite, peak nitrate, and peak acetate concentrations in plasma. Abbreviations: INU, inulin; NO_3_^−^, nitrate. ‘rs’ indicates Spearman’s rank correlation coefficient, ‘r’ indicates Pearson’s correlation coefficient. *p < 0.05, ** *p* < 0.01.

## Discussion

This study represents the first double-blind crossover investigation examining the direct impacts of acute dietary intervention with inorganic nitrate and inulin, focusing on their potential synergistic effects on their derived metabolites. Based on prior preclinical research (Catry et al., 2018), an *in vitro* study (Sakakibara et al., 2010), and the understanding that nitrate supplementation enhances NO bioavailability via the nitrate-nitrite-NO pathway (Lundberg et al., 2018), we hypothesised that combining inulin and nitrate might increase NO bioavailability, as indicated by elevated plasma nitrate and nitrite concentrations. However, combined inulin and nitrate did not elevate plasma nitrate or nitrite concentrations beyond those observed with nitrate alone. Plasma nitrate and nitrite increased following nitrate but remained unchanged with inulin ingestion, suggesting that neither inulin nor its metabolite acetate significantly influenced NO bioavailability in humans. Inulin has been proposed as a dietary approach to increase the production of SCFAs, including acetate (Boets et al., 2015), potentially exceeding the levels observed with other indigestible carbohydrates (van der Beek et al., 2018). Nevertheless, in this study, we observed only modest increases in plasma acetate, as well as butyrate and propionate, within a 360-minute timeframe. This lack of a distinct peak is likely attributable to the limited duration of supplementation. Additionally, while reductions in DBP and MAP from baseline were observed, no significant differences were found across the different conditions. Nonetheless, significant inverse relationships were identified between DBP, MAP and plasma nitrite following combined inulin and nitrate supplementation, suggesting potential interactive effects on BP. A similar inverse association was also noted between nitrate intake and DBP, corroborating findings from previous research (Wei et al., 2023; Wei et al., 2024).

### Plasma nitrate and nitrite

After nitrate ingestion, plasma nitrate and nitrite concentrations increase via the nitrate-nitrite-NO pathway, peaking at 1–2 hours and 2–3 hours, respectively, before gradually declining and returning to baseline levels within 24 hours (Webb et al., 2008). Various enzymes and proteins, including deoxyhaemoglobin, can catalyse the conversion of nitrite to NO within the blood and other tissues (Cosby et al., 2003). Consistent with previous research on adults with normal BP, hypertension, and obesity (Webb et al., 2008; Wylie et al., 2013; Kapil et al., 2015), this study observed increases in plasma nitrate and nitrite after nitrate supplementation, both with and without inulin. After supplementation, nitrate peaked at 60 minutes and nitrite at 120 minutes, with elevated concentrations persisting for up to 360 minutes. No differences in peak timing were observed between combined inulin and nitrate and nitrate alone, indicating that their combination does not alter the pharmacokinetics of nitrate or nitrite, as seen with other prebiotics (Rodriguez-Mateos et al., 2015).

Nitrate and inulin have been suggested to enhance NO bioavailability via distinct mechanisms. However, unlike nitrate, the effects of which have been documented in clinical studies (Wei et al., 2024), the effects of inulin have only been demonstrated in preclinical (Catry et al., 2018) and *in vitro* (Sakakibara et al., 2010) studies, with no clinical trials conducted in humans to date. Previous *in vivo* studies have shown that the dietary context can modulate the effects of inorganic nitrate on NO metabolism, particularly when co-ingested with prebiotic compounds that influence eNOS phosphorylation (Lovegrove et al., 2017; Álvarez-Cilleros et al., 2018). However, the combination of apples (a source of flavonoids) with spinach (a source of nitrate) did not enhance NO bioavailability, but instead attenuated SBP responses (Bondonno et al., 2012). Similarly, cocoa flavanols, another prebiotic-rich food, markedly reduced plasma nitrite concentrations when consumed with dietary nitrate (Rodriguez-Mateos et al., 2015), an effect we did not observe with inulin and nitrate co-supplementation. The relationship between plasma nitrite levels and eNOS phosphorylation is complex. While elevated plasma nitrite concentrations suggest enhanced NO availability and eNOS activity under normal physiological conditions (Lauer et al., 2001; Kleinbongard et al., 2003), others have proposed that dietary nitrate-derived plasma nitrite increases, while eNOS-derived nitrite might decrease, resulting in unchanged plasma nitrite concentrations. This supports the hypothesis that activation of the nitrate–nitrite–NO pathway downregulates eNOS activity (Carlström et al., 2015). Although we did not observe changes in plasma nitrite levels, we cannot exclude the possibility that inulin and nitrate supplementation influenced eNOS phosphorylation, as this was not directly evaluated.

### Plasma short-chain fatty acids

Inulin is not absorbed until it reaches the colon, where it is fermented into SCFAs by the gut bacteria. Studies have shown that inulin consumption can elevate plasma SCFAs concentrations within hours (Tarini and Wolever, 2010; Boets et al., 2015; van der Beek et al., 2018). We observed that inulin intake resulted in higher plasma acetate, butyrate, and propionate concentrations, with and without nitrate, than with nitrate alone. However, these increases were not statistically significant when baseline values were excluded from the iAUC analysis, possibly because of individual variations in postprandial SCFAs responses. Our findings partly align with those of Fernandes et al. (2011), who observed a significant increase in breath hydrogen and methane at 120 minutes following inulin consumption (24 g) and only observed a non-significant trend in serum SCFAs up to 240 minutes post-consumption. However, our findings contrast with those of other studies that reported a significant increase in SCFAs after acute inulin consumption.For example, Van der Beek et al. (2018) found a significant rise in plasma acetate 240–420 minutes post-ingestion compared to maltodextrin. Similarly, Tarini and Wolever (2010) found that 24 g of inulin significantly increased all three serum SCFAs within a 6-hour period, while Rahat-Rozenbloom et al. (2017) showed an increase in SCFAs 240–360 minutes after 24 g of inulin intake. The discrepancies between our findings and those of previous studies may stem from differences in the types of inulin used. Van der Beek et al. (2018) used short-chain inulin, leading to rapid fermentation and elevated SCFAs at 240 minutes post-ingestion. In contrast, we used long-chain inulin, which results in slower fermentation (Stewart et al., 2008). Our inulin dose was 9 g lower than the amounts used in most studies to limit common side effects with higher doses of this supplement (i.e., bloating, flatulence) (Bonnema et al., 2010), which may have affected timing and quantities of SCFAs production, as inulin effects are dose-dependent (Vinelli et al., 2022). Studies using 15 g inulin found increases in all SCFAs at 12 hours post-consumption (Boets et al., 2015). By measuring only up to 6 hours (360 minutes) post-consumption, we might have missed interindividual variability on transit time, meaning a further increase in SCFAs after 6 hours in those participants with a slower digestion.

The impact of dietary nitrate on the gut microbiome and SCFAs is not yet well understood. For instance, Wang et al. (2023) discovered that while nitrate supplementation through red beetroot juice does not seem to affect alpha and beta diversity, it does lead to significant changes in the abundance of certain taxa, such as *Romboutsia*, *Bacteroidales*, and *Akkermansia muciniphila*, after a 14-day supplementation period. In contrast, Messiha et al. (2025) reported mixed outcomes with nitrate supplementation in the form of sodium nitrate, which modified the gut microbiome and elevated proatherogenic metabolites such as trimethylamine N-oxide (TMAO). In the latter study, the authors also observed an increase in *A. muciniphila* compared to placebo, suggesting a compensatory response to elevated TMAO levels, as this bacterium can reduce TMAO. Additionally, the authors noted an increase in *Clostridiales*, which contribute to TMAO production (Messiha et al., 2025). However, much like our findings, where treatment differences were likely due to inulin fermentation into SCFAs, Messiha et al. (2025) showed no significant reduction in plasma SCFAs (acetate, propionate, butyrate, and caproate) following nitrate supplementation.

### Blood pressure

Nitrate acts as a reservoir for NO and reduces BP through vasodilation via the sGC–cGMP pathway, which decreases reactive oxygen species, inhibits oxidative stress enzymes, and enhances eNOS function (Carlström et al., 2018). Despite an increase in plasma nitrate and nitrite concentrations, we observed no significant differences in BP between conditions. This finding contrasts with those of some studies (Kapil et al., 2010; Vanhatalo et al., 2010) but aligns with others (Miller et al., 2012; Wei et al., 2023). Studies have demonstrated BP reductions with nitrate supplementation in adults with normal BP (Kapil et al., 2010; Bahra et al., 2012; Wei et al., 2023) and hypertension (Ghosh et al., 2013; Kapil et al., 2015). However, nitrate does not consistently lower BP, even when plasma nitrate and nitrite concentrations are elevated (Bescos et al., 2025). Baseline BP seems to influence the BP reduction achieved with nitrate supplementation (Kapil et al., 2010), with supplementation potentially being more effective in older adults with BMI > 30 kg/m^2^ or prehypertension (He et al., 2021). Although we observed no reduction in BP, our findings revealed correlations between changes in peak nitrite and DBP and MAP following combined inulin and nitrate supplementation, and peak nitrate and DBP following nitrate supplementation alone, potentially suggesting individual variations in BP responses linked to NO bioavailability. These interindividual differences support the concept of higher and lower responses to nitrate supplementation (Hayes et al., 2025).

### Strengths and limitations

The strengths of this study include its crossover design, which effectively controlled for baseline differences, and the assessment of plasma SCFAs rather than faecal SCFAs, which provides a representative measure of systemic circulation (den Besten et al., 2013). In addition, this study controlled for dietary nitrate intake. However, this study has several limitations that warrant consideration. The young, healthy study population may not be representative of the general population and may have a limited potential for BP improvement. Additionally, the analysis did not account for habitual fibre intake, which could have influenced the results. Future studies should consider participants’ regular fibre intake and dietary history to contextualise their responses to these interventions, offering a more comprehensive understanding of the potential benefits (Whelan et al., 2024). Moreover, the 360-minute post-intervention blood collection may have failed to capture peak SCFAs concentrations in some participants due to variability in gut microbiota fibre fermentation rates, attributable to diverse bacterial metabolite profiles and gut physiology, among other factors (Thomson et al., 2021). In this study, BP was assessed using office-based measurements, which are not regarded as the gold standard in comparison to ambulatory BP monitoring (Asayama et al., 2024). Lastly, the absence of a placebo control and the small sample size limit the ability to distinguish supplement effects from natural variations, reducing the statistical power to identify significant differences, which could affect the generalisability of the findings.

### Future perspectives

Investigating the chronic consumption of inulin and nitrate is essential to understanding their combined health effects in real-world dietary contexts, as individuals consume foods rather than isolated compounds. This approach provides a realistic assessment of potential health benefits. Long-term studies on the synergistic effects of inulin and nitrate may elucidate their impact on vascular function, gut microbiome composition, and cardiovascular health, particularly in populations at an elevated risk of cardiovascular diseases. Such research should prioritise assessing the effectiveness of these supplements in reducing BP in adults with hypertension, rather than those with normal BP. For example, elevation in plasma nitrite concentration has been demonstrated to be significantly greater in older individuals than in their younger counterparts (Stanaway et al., 2019), indicating that enhancement of the enterosalivary pathway may potentially result in unexpectedly more favourable outcomes with respect to cardiovascular parameters in the older population. Additionally, it is crucial to examine the impact of inulin consumption in older adults, who may respond differently to age-related changes in the gut microbiota (Kiewiet et al., 2021). For instance, older individuals with reduced caloric needs may require foods enriched with fibre or the use of fibre supplements (McKeown et al., 2022).

## Conclusion

In the short term, acute supplementation with inulin and nitrate did not demonstrate any additional effect on plasma nitrate and nitrite levels compared to nitrate supplementation alone. Similarly, nitrate supplementation did not appear to adversely affect plasma SCFAs; however, a longer duration may be required to observe inulin-derived plasma SCFAs. BP was not significantly influenced by the supplements; nonetheless, significant negative correlations were identified between peak plasma nitrite and both DBP and MAP for the combined supplementation, as well as between peak plasma nitrate and DBP following nitrate supplementation alone. Consequently, further research is necessary to investigate the effects of chronic inulin and nitrate supplementation in adults with hypertension, with the aim of evaluating the impact on BP, vascular function, and gut microbiota composition, while considering variations in dietary history and fibre intake.

## Supporting information

Virgili et al. supplementary material Virgili et al. supplementary material

## Supplementary material

The supplementary material for this article can be found at http://doi.org/10.1017/gmb.2025.10008.

## References

[r1] Aldubayan MA, Mao X, Laursen MF, Pigsborg K, Christensen LH, Roager HM, Nielsen DS, Hjorth MF and Magkos F (2023) Supplementation with inulin-type fructans affects gut microbiota and attenuates some of the cardiometabolic benefits of a plant-based diet in individuals with overweight or obesity. Frontiers in Nutrition 10, 1108088. 10.3389/fnut.2023.1108088. 37181156 PMC10167298

[r2] Álvarez-Cilleros D, Ramos S, Goya L and Martín M (2018) Colonic metabolites from flavanols stimulate nitric oxide production in human endothelial cells and protect against oxidative stress-induced toxicity and endothelial dysfunction. Food and Chemical Toxicology 115, 88–97. 10.1016/j.fct.2018.03.006. 29530637

[r3] Asayama K, Ohkubo T and Imai Y (2024) In-office and out-of-office blood pressure measurement. Journal of Human Hypertension 38(6), 477–485. 10.1038/s41371-021-00486-8. 33785904 PMC8008215

[r4] Ashworth A and Bescos R (2017) Dietary nitrate and blood pressure: Evolution of a new nutrient? Nutrition Research Reviews 30(2), 208–219. 10.1017/S0954422417000063. 28511731

[r5] Azuma N, Saito Y, Nishijima T, Aoki R and Nishihira J (2023) Effect of daily ingestion of Bifidobacterium and dietary fiber on vascular endothelial function: A randomized, double-blind, placebo-controlled, parallel-group comparison study. Bioscience, Biotechnology, and Biochemistry 88(1), 86–96. 10.1093/bbb/zbad148. 37849220

[r6] Bahadoran Z, Mirmiran P, Kabir A, Azizi F and Ghasemi A (2017) The nitrate-independent blood pressure–lowering effect of beetroot juice: A systematic review and meta-analysis. Advances in Nutrition 8(6), 830–838. 10.3945/an.117.016717. 29141968 PMC5683004

[r7] Bahra M, Kapil V, Pearl V, Ghosh S and Ahluwalia A (2012) Inorganic nitrate ingestion improves vascular compliance but does not alter flow-mediated dilatation in healthy volunteers. Nitric Oxide 26(4), 197–202. 10.1016/j.niox.2012.01.004. 22285857 PMC3405527

[r8] van der Beek CM, Canfora EE, Kip AM, Gorissen SHM, Olde Damink SWM, van HM, Holst JJ, Blaak EE, Dejong CHC and Lenaerts K (2018) The prebiotic inulin improves substrate metabolism and promotes short-chain fatty acid production in overweight to obese men. Metabolism 87, 25–35. 10.1016/j.metabol.2018.06.009. 29953876

[r9] Bescos R, Gallardo-Alfaro L, Ashor A, Rizzolo-Brime L, Siervo M and Casas-Agustench P (2025) Nitrate and nitrite bioavailability in plasma and saliva: Their association with blood pressure — A systematic review and meta-analysis. Free Radical Biology and Medicine 226, 70–83. 10.1016/j.freeradbiomed.2024.11.010. 39522567

[r10] den Besten G, van Eunen K, Groen AK, Venema K, Reijngoud DJ and Bakker BM (2013) The role of short-chain fatty acids in the interplay between diet, gut microbiota, and host energy metabolism. Journal of Lipid Research 54(9), 2325–2340. 10.1194/jlr.R036012. 23821742 PMC3735932

[r11] Blanca MJ, Arnau J, García-Castro FJ, Alarcón R and Bono R (2023) Non-normal data in repeated measures ANOVA: Impact on type I error and power. Psicothema 35(1), 21–29. 10.7334/psicothema2022.292. 36695847

[r12] Boets E, Deroover L, Houben E, Vermeulen K, Gomand SV, Delcour JA and Verbeke K (2015) Quantification of in vivo colonic short chain fatty acid production from inulin. Nutrients 7(11), 8916–8929. 10.3390/nu7115440. 26516911 PMC4663568

[r13] Bondonno CP, Yang X, Croft KD, Considine MJ, Ward NC, Rich L, Puddey IB, Swinny E, Mubarak A and Hodgson JM (2012) Flavonoid-rich apples and nitrate-rich spinach augment nitric oxide status and improve endothelial function in healthy men and women: A randomized controlled trial. Free Radical Biology and Medicine 52(1), 95–102. 10.1016/j.freeradbiomed.2011.09.028. 22019438

[r14] Bonnema AL, Kolberg LW, Thomas W and Slavin JL (2010) Gastrointestinal tolerance of chicory inulin products. Journal of the American Dietetic Association 110(6), 865–868. 10.1016/j.jada.2010.03.025. 20497775

[r15] Capper TE, Siervo M, Clifford T, Taylor G, Iqbal W, West D and Stevenson EJ (2022) Pharmacokinetic profile of incremental Oral doses of dietary nitrate in young and older adults: A crossover randomized clinical trial. The Journal of Nutrition 152(1), 130–139. 10.1093/jn/nxab354. 34718635 PMC8754575

[r16] Carlström M, Liu M, Yang T, Zollbrecht C, Huang L, Peleli M, Borniquel S, Kishikawa H, Hezel M, Persson AE, Weitzberg E and Lundberg JO (2015) Cross-talk between nitrate-nitrite-NO and NO synthase pathways in control of vascular NO homeostasis. Antioxidants & Redox Signaling 23(4), 295–306. 10.1089/ars.2013.5481. 24224525 PMC4523008

[r17] Carlström M, Lundberg JO and Weitzberg E (2018) Mechanisms underlying blood pressure reduction by dietary inorganic nitrate. Acta Physiologica (Oxford, England) 224(1), e13080. 10.1111/apha.13080. 29694703

[r18] Catry E, Bindels LB, Tailleux A, Lestavel S, Neyrinck AM, Goossens JF, Lobysheva I, Plovier H, Essaghir A, Demoulin JB, Bouzin C, Pachikian BD, Cani PD, Staels B, Dessy C and Delzenne NM (2018) Targeting the gut microbiota with inulin-type fructans: Preclinical demonstration of a novel approach in the management of endothelial dysfunction. Gut 67(2), 271–283. 10.1136/gutjnl-2016-313316. 28377388 PMC5868295

[r19] Clemente-Suárez VJ, Beltrán-Velasco AI, Redondo-Flórez L, Martín-Rodríguez A and Tornero-Aguilera JF (2023) Global impacts of Western diet and its effects on metabolism and health: A narrative review. Nutrients 15(12), 2749. 10.3390/nu15122749. 37375654 PMC10302286

[r20] Cosby K, Partovi KS, Crawford JH, Patel RP, Reiter CD, Martyr S, Yang BK, Waclawiw MA, Zalos G, Xu X, Huang KT, Shields H, Kim-Shapiro DB, Schechter AN, Cannon RO and Gladwin MT (2003) Nitrite reduction to nitric oxide by deoxyhemoglobin vasodilates the human circulation. Nature Medicine 9(12), 1498–1505. 10.1038/nm954. 14595407

[r21] Depeint F, Tzortzis G, Vulevic J, I’Anson K and Gibson GR (2008) Prebiotic evaluation of a novel galactooligosaccharide mixture produced by the enzymatic activity of Bifidobacterium bifidum NCIMB 41171, in healthy humans: A randomized, double-blind, crossover, placebo-controlled intervention study2. The American Journal of Clinical Nutrition 87(3), 785–791. 10.1093/ajcn/87.3.785. 18326619

[r72] Dei Cas, M., Paroni, R., Saccardo, A., Casagni, E., Arnoldi, S., Gambaro, V., Saresella, M., Mario, C., La Rosa, F., Marventano, I., Piancone, F., & Roda, G. (2020). A straightforward LC-MS/MS analysis to study serum profile of short and medium chain fatty acids. Journal of chromatography. B, Analytical technologies in the biomedical and life sciences, 1154, 121982. 10.1016/j.jchromb.2020.121982 32862023

[r22] Fernandes J, Vogt J and Wolever TM (2011) Inulin increases short-term markers for colonic fermentation similarly in healthy and hyperinsulinaemic humans. European Journal of Clinical Nutrition 65(12), 1279–1286. 10.1038/ejcn.2011.116. 21712835 PMC3937120

[r23] Ghosh SM, Kapil V, Fuentes-Calvo I, Bubb KJ, Pearl V, Milsom AB, Khambata R, Maleki-Toyserkani S, Yousuf M, Benjamin N, Webb AJ, Caulfield MJ, Hobbs AJ and Ahluwalia A (2013) Enhanced vasodilator activity of nitrite in hypertension: Critical role for erythrocytic xanthine oxidoreductase and translational potential. Hypertension 61(5), 1091–1102. 10.1161/hypertensionaha.111.00933. 23589565

[r24] González-Soltero R, Bailén M, de Lucas B, Ramírez-Goercke MI, Pareja-Galeano H and Larrosa M (2020) Role of Oral and gut microbiota in dietary nitrate metabolism and its impact on sports performance. Nutrients 12(12), 3611. 10.3390/nu12123611. 33255362 PMC7760746

[r25] Hayes E, Alhulaefi S, Siervo M, Whyte E, Kimble R, Matu J, Griffiths A, Sim M, Burleigh M, Easton C, Lolli L, Atkinson G, Mathers JC and Shannon OM (2025) Inter-individual differences in the blood pressure lowering effects of dietary nitrate: A randomised double-blind placebo-controlled replicate crossover trial. European Journal of Nutrition 64(2), 101. 10.1007/s00394-025-03616-x. 39992469 PMC11850510

[r26] He Y, Liu J, Cai H, Zhang J, Yi J, Niu Y, Xi H, Peng X and Guo L (2021) Effect of inorganic nitrate supplementation on blood pressure in older adults: A systematic review and meta-analysis. Nitric Oxide 113–114, 13–22. 10.1016/j.niox.2021.04.006. 33905826

[r27] Jakubcik EM, Rutherfurd-Markwick K, Chabert M, Wong M and Ali A (2021) Pharmacokinetics of nitrate and nitrite following beetroot juice drink consumption. Nutrients 13(2), 281. 10.3390/nu13020281. 33498220 PMC7908977

[r28] Jama HA, Rhys-Jones D, Nakai M, Yao CK, Climie RE, Sata Y, Anderson D, Creek DJ, Head GA, Kaye DM, Mackay CR, Muir J and Marques FZ (2023) Prebiotic intervention with HAMSAB in untreated essential hypertensive patients assessed in a phase II randomized trial. Nature Cardiovascular Research 2(1), 35–43. 10.1038/s44161-022-00197-4. 39196205

[r29] Jama HA, Snelson M, Schutte AE, Muir J and Marques FZ (2024) Recommendations for the use of dietary Fiber to improve blood pressure control. Hypertension 81(7), 1450–1459. 10.1161/HYPERTENSIONAHA.123.22575. 38586958

[r30] Kapil V, Milsom AB, Okorie M, Maleki-Toyserkani S, Akram F, Rehman F, Arghandawi S, Pearl V, Benjamin N, Loukogeorgakis S, Macallister R, Hobbs AJ, Webb AJ and Ahluwalia A (2010) Inorganic nitrate supplementation lowers blood pressure in humans: Role for nitrite-derived NO. Hypertension 56(2), 274–281. 10.1161/hypertensionaha.110.153536. 20585108

[r31] Kapil V, Khambata RS, Robertson A, Caulfield MJ and Ahluwalia A (2015) Dietary nitrate provides sustained blood pressure lowering in hypertensive patients: A randomized, phase 2, double-blind, placebo-controlled study. Hypertension 65(2), 320–327. 10.1161/hypertensionaha.114.04675. 25421976 PMC4288952

[r32] Kario K, Okura A, Hoshide S and Mogi M (2024) The WHO global report 2023 on hypertension warning the emerging hypertension burden in globe and its treatment strategy. Hypertension Research 47(5), 1099–1102. 10.1038/s41440-024-01622-w. 38443614

[r33] Kaur AP, Bhardwaj S, Dhanjal DS, Nepovimova E, Cruz-Martins N, Kuča K, Chopra C, Singh R, Kumar H, Șen F, Kumar V, Verma R and Kumar D (2021) Plant prebiotics and their role in the amelioration of diseases. Biomolecules 11(3), 440. 10.3390/biom11030440. 33809763 PMC8002343

[r34] Kaye DM, Shihata WA, Jama HA, Tsyganov K, Ziemann M, Kiriazis H, Horlock D, Vijay A, Giam B, Vinh A, Johnson C, Fiedler A, Donner D, Snelson M, Coughlan MT, Phillips S, Du XJ, El-Osta A, Drummond G, … Marques FZ (2020) Deficiency of prebiotic Fiber and insufficient Signaling through gut metabolite-sensing receptors leads to cardiovascular disease. Circulation 141(17), 1393–1403. 10.1161/CIRCULATIONAHA.119.043081 32093510

[r35] Kiewiet MBG, Elderman ME, El Aidy S, Burgerhof JGM, Visser H, Vaughan EE, Faas MM and de Vos P (2021) Flexibility of gut microbiota in ageing individuals during dietary Fiber long-chain inulin intake. Molecular Nutrition & Food Research 65(4), e2000390. 10.1002/mnfr.202000390. 33369019 PMC8138623

[r36] Kleinbongard P, Dejam A, Lauer T, Rassaf T, Schindler A, Picker O, Scheeren T, Gödecke A, Schrader J, Schulz R, Heusch G, Schaub GA, Bryan NS, Feelisch M and Kelm M (2003) Plasma nitrite reflects constitutive nitric oxide synthase activity in mammals. Free Radical Biology and Medicine 35(7), 790–796. 10.1016/S0891-5849(03)00406-4. 14583343

[r37] Lauer T, Preik M, Rassaf T, Strauer BE, Deussen A, Feelisch M and Kelm M (2001) Plasma nitrite rather than nitrate reflects regional endothelial nitric oxide synthase activity but lacks intrinsic vasodilator action. Proceedings of the National Academy of Sciences of the United States of America 98(22), 12814–12819. 10.1073/pnas.221381098. 11606734 PMC60136

[r38] Li D, Jovanovski E, Zurbau A, Sievenpiper J, Milicic D, El-Sohemy A and Vuksan V (2024) No difference between the efficacy of high-nitrate and low-nitrate vegetable supplementation on blood pressure after 16 weeks in individuals with early-stage hypertension: An exploratory, double-blinded, randomized, controlled trial. Nutrients 16(17), 3018. 10.3390/nu16173018. 39275333 PMC11397180

[r39] Lovegrove JA, Stainer A and Hobbs DA (2017) Role of flavonoids and nitrates in cardiovascular health. Proceedings of the Nutrition Society 76(2), 83–95. 10.1017/S0029665116002871. 28100284

[r40] Lundberg JO and Weitzberg E (2005) NO generation from nitrite and its role in vascular control. Arteriosclerosis, Thrombosis, and Vascular Biology 25(5), 915–922. 10.1161/01.ATV.0000161048.72004.c2. 15746440

[r41] Lundberg JO, Weitzberg E and Gladwin MT (2008) The nitrate-nitrite-nitric oxide pathway in physiology and therapeutics. Nature Reviews. Drug Discovery 7(2), 156–167. 10.1038/nrd2466. 18167491

[r42] Lundberg JO, Carlström M and Weitzberg E (2018) Metabolic effects of dietary nitrate in health and disease. Cell Metabolism 28(1), 9–22. 10.1016/j.cmet.2018.06.007. 29972800

[r43] McDonagh STJ, Wylie LJ, Webster JMA, Vanhatalo A and Jones AM (2018) Influence of dietary nitrate food forms on nitrate metabolism and blood pressure in healthy normotensive adults. Nitric Oxide 72, 66–74. 10.1016/j.niox.2017.12.001. 29223585

[r44] McEvoy JW, McCarthy CP, Bruno RM, Brouwers S, Canavan MD, Ceconi C, Christodorescu RM, Daskalopoulou SS, Ferro CJ, Gerdts E, Hanssen H, Harris J, Lauder L, McManus RJ, Molloy GJ, Rahimi K, Regitz-Zagrosek V, Rossi GP, Sandset EC and Touyz RM (2024) 2024 ESC guidelines for the management of elevated blood pressure and hypertension. European Heart Journal 45(38), 3912–4018. 10.1093/eurheartj/ehae178. 39210715

[r45] McKeown NM, Fahey GC, Slavin and van der Kamp JW (2022) Fibre intake for optimal health: How can healthcare professionals support people to reach dietary recommendations? BMJ 378, e054370. 10.1136/bmj-2020-054370. 35858693 PMC9298262

[r46] Messiha D, Rinke M, Schultz Moreira Amos A, Tratnik A, Hendgen-Cotta UB, Lortz J, Hogrebe K, Kehrmann J, Buer J, Rassaf T and Rammos C (2025) Influence of chronic dietary nitrate on downstream Atherogenic metabolites and the enteral microbiome—A double-blind randomized controlled trial. Dietetics 4(1), 1. https://www.mdpi.com/2674-0311/4/1/1

[r47] Miller GD, Marsh AP, Dove RW, Beavers D, Presley T, Helms C, Bechtold E, King SB and Kim-Shapiro D (2012) Plasma nitrate and nitrite are increased by a high-nitrate supplement but not by high-nitrate foods in older adults. Nutrition Research 32(3), 160–168. 10.1016/j.nutres.2012.02.002. 22464802 PMC3319660

[r48] Mishra SR, Satheesh G, Khanal V, Nguyen TN, Picone D, Chapman N and Lindley RI (2025) Closing the gap in global disparities in hypertension control. Hypertension 82(3), 407–410. 10.1161/HYPERTENSIONAHA.124.24137. 39970253

[r49] Mukaka MM (2012) Statistics corner: A guide to appropriate use of correlation coefficient in medical research. Malawi Medical Journal 24(3), 69–71. https://www.ncbi.nlm.nih.gov/pmc/articles/PMC3576830/pdf/MMJ2403-0069.pdf 23638278 PMC3576830

[r69] Muntner, P., Shimbo, D., Carey, R. M., Charleston, J. B., Gaillard, T., Misra, S., Myers, M. G., Ogedegbe, G., Schwartz, J. E., Townsend, R. R., Urbina, E. M., Viera, A. J., White, W. B., & Wright, J. T., Jr (2019). Measurement of Blood Pressure in Humans: A Scientific Statement From the American Heart Association. Hypertension (Dallas, Tex: 1979). 73(5), e35–e66. 10.1161/HYP.0000000000000087 30827125 PMC11409525

[r50] Norouzzadeh M, Hasan Rashedi M, Ghaemi S, Saber N, Mirdar Harijani A, Habibi H, Mostafavi S, Sarv F, Farhadnejad H, Teymoori F, Khaleghian M and Mirmiran P (2025) Plasma nitrate, dietary nitrate, blood pressure, and vascular health biomarkers: A GRADE-assessed systematic review and dose-response meta-analysis of randomized controlled trials. Nutrition Journal 24(1), 47. 10.1186/s12937-025-01114-8. 40128734 PMC11931885

[r71] Piknova, B., Park, J. W., Cassel, K. S., Gilliard, C. N., & Schechter, A. N. (2016). Measuring Nitrite and Nitrate, Metabolites in the Nitric Oxide Pathway, in Biological Materials using the Chemiluminescence Method. J Vis Exp(118), 54879. 10.3791/54879 PMC5226469 28060334

[r51] Rahat-Rozenbloom S, Fernandes J, Cheng J and Wolever TMS (2017) Acute increases in serum colonic short-chain fatty acids elicited by inulin do not increase GLP-1 or PYY responses but may reduce ghrelin in lean and overweight humans. European Journal of Clinical Nutrition 71(8), 953–958. 10.1038/ejcn.2016.249. 27966574 PMC5423780

[r52] Rodriguez-Mateos A, Hezel M, Aydin H, Kelm M, Lundberg JO, Weitzberg E, Spencer JP and Heiss C (2015) Interactions between cocoa flavanols and inorganic nitrate: Additive effects on endothelial function at achievable dietary amounts. Free Radical Biology & Medicine 80, 121–128. 10.1016/j.freeradbiomed.2014.12.009. 25530151

[r53] Sakakibara S, Murakami R, Takahashi M, Fushimi T, Murohara T, Kishi M, Kajimoto Y, Kitakaze M and Kaga T (2010) Vinegar intake enhances flow-mediated vasodilatation via upregulation of endothelial nitric oxide synthase activity. Bioscience, Biotechnology, and Biochemistry 74(5), 1055–1061. 10.1271/bbb.90953. 20460711

[r54] Siervo M, Lara J, Ogbonmwan I and Mathers JC (2013) Inorganic nitrate and beetroot juice supplementation reduces blood pressure in adults: A systematic review and meta-analysis. The Journal of Nutrition 143(6), 818–826. 10.3945/jn.112.170233. 23596162

[r55] Stanaway L, Rutherfurd-Markwick K, Page R, Wong M, Jirangrat W, Teh KH and Ali A (2019) Acute supplementation with nitrate-Rich beetroot juice causes a greater increase in plasma nitrite and reduction in blood pressure of older compared to younger adults. Nutrients 11(7). 10.3390/nu11071683. PMC6683255 31336633

[r56] Stewart ML, Timm DA and Slavin JL (2008) Fructooligosaccharides exhibit more rapid fermentation than long-chain inulin in an in vitro fermentation system. Nutrition Research 28(5), 329–334. 10.1016/j.nutres.2008.02.014. 19083428

[r57] Tarini J and Wolever TM (2010) The fermentable fibre inulin increases postprandial serum short-chain fatty acids and reduces free-fatty acids and ghrelin in healthy subjects. Applied Physiology, Nutrition, and Metabolism 35(1), 9–16. 10.1139/h09-119. 20130660

[r58] Thomson C, Garcia AL and Edwards CA (2021) Interactions between dietary fibre and the gut microbiota. Proceedings of the Nutrition Society 80(4), 398–408. 10.1017/S0029665121002834. 34551829

[r59] Vanhatalo A, Bailey SJ, Blackwell JR, DiMenna FJ, Pavey TG, Wilkerson DP, Benjamin N, Winyard PG and Jones AM (2010) Acute and chronic effects of dietary nitrate supplementation on blood pressure and the physiological responses to moderate-intensity and incremental exercise. American Journal of Physiology. Regulatory, Integrative and Comparative Physiology 299(4), R1121–R1131. 10.1152/ajpregu.00206.2010. 20702806

[r60] Vinelli V, Biscotti P, Martini D, Del Bo C, Marino M, Meroño T, Nikoloudaki O, Calabrese FM, Turroni S, Taverniti V, Unión Caballero A, Andrés-Lacueva C, Porrini M, Gobbetti M, De Angelis M, Brigidi P, Pinart M, Nimptsch K, Guglielmetti S and Riso P (2022) Effects of dietary Fibers on short-chain fatty acids and gut microbiota composition in healthy adults: A systematic review. Nutrients 14(13), 2559. 10.3390/nu14132559. 35807739 PMC9268559

[r61] Wang Y, Do T, Marshall LJ and Boesch C (2023) Effect of two-week red beetroot juice consumption on modulation of gut microbiota in healthy human volunteers – A pilot study. Food Chemistry 406, 134989. 10.1016/j.foodchem.2022.134989. 36527987

[r62] Webb AJ, Patel N, Loukogeorgakis S, Okorie M, Aboud Z, Misra S, Rashid R, Miall P, Deanfield J, Benjamin N, MacAllister R, Hobbs AJ and Ahluwalia A (2008) Acute blood pressure lowering, vasoprotective, and antiplatelet properties of dietary nitrate via bioconversion to nitrite. Hypertension 51(3), 784–790. 10.1161/hypertensionaha.107.103523. 18250365 PMC2839282

[r63] Wei C, Vanhatalo A, Kadach S, Stoyanov Z, Abu-Alghayth M, Black MI, Smallwood MJ, Rajaram R, Winyard PG and Jones AM (2023) Reduction in blood pressure following acute dietary nitrate ingestion is correlated with increased red blood cell S-nitrosothiol concentrations. Nitric Oxide 138–139, 1–9. 10.1016/j.niox.2023.05.008. 37268184

[r64] Wei C, Vanhatalo A, Black MI, Blackwell JR, Rajaram R, Kadach S and Jones AM (2024) Relationships between nitric oxide biomarkers and physiological outcomes following dietary nitrate supplementation. Nitric Oxide 148, 23–33. 10.1016/j.niox.2024.04.010. 38697467

[r65] Whelan K, Alexander M, Gaiani C, Lunken G, Holmes A, Staudacher HM, Theis S and Marco ML (2024) Design and reporting of prebiotic and probiotic clinical trials in the context of diet and the gut microbiome. Nature Microbiology 9(11), 2785–2794. 10.1038/s41564-024-01831-6. 39478082

[r66] Whitehead AL, Julious SA, Cooper CL and Campbell MJ (2016) Estimating the sample size for a pilot randomised trial to minimise the overall trial sample size for the external pilot and main trial for a continuous outcome variable. Statistical Methods in Medical Research 25(3), 1057–1073. 10.1177/0962280215588241. 26092476 PMC4876429

[r67] Wylie LJ, Kelly J, Bailey SJ, Blackwell JR, Skiba PF, Winyard PG, Jeukendrup AE, Vanhatalo A and Jones AM (2013) Beetroot juice and exercise: Pharmacodynamic and dose-response relationships. Journal of Applied Physiology (1985) 115(3), 325–336. 10.1152/japplphysiol.00372.2013 23640589

[r68] Xu J, Moore BN and Pluznick JL (2022) Short-chain fatty acid receptors and blood pressure regulation: Council on hypertension mid-career award for research excellence 2021. Hypertension 79(10), 2127–2137. 10.1161/hypertensionaha.122.18558. 35912645 PMC9458621

