# The effects of inorganic nitrate and inulin co-ingestion on circulating metabolites and blood pressure in young adults: a pilot double-blind randomised crossover trial – CORRIGENDUM

**DOI:** 10.1017/gmb.2025.10010

**Published:** 2025-09-08

**Authors:** Jessica Virgili, Gwenaelle Le Gall, Anni Vanhatalo, Bert Bond, David Vauzour, Luciana Torquati

The author regrets the inclusion of two errors in their article. The first error is the incorrect *p*-value in the abstract. The *p*-value reported for the correlation between diastolic blood pressure following nitrate supplementation (*r*ₛ = −0.47, *p* = 0.004) should be *p* = 0.04. This has been updated in the original article.

The second error is the inclusion of incorrectly rounded values displayed in Table 4. The correct Table 4 is displayed below:

**Table 4.** Correlation coefficients of peak changes in plasma nitrite, nitrate, and acetate with their corresponding blood pressure variables following acute ingestion of the three supplements

Metabolites: Supplement(s)	ΔSBP	ΔDBP	ΔMAP
Δ Peak plasma nitrite (NO_2_^−^): INU + NO_3_^−^	*r*_s_ = 0.01, *p* = 0.955	***r***_**s**_ = −0.61, *p* = 0.004**	***r***_**s**_ = −0.59, *p* = 0.005**
Δ Peak plasma nitrite (NO_2_^−^): NO_3_^−^	*r*_s_ = −0.41, *p* = 0.06	*r*_s_ = −0.13, *p* = 0.56	*r*_s_ = −0.29, *p* = 0.20
Δ Peak plasma nitrate (NO_3_^−^): INU + NO_3_^−^	*r*_s_ = 0.29, *p* = 0.22	*r*_s_ = 0.13, *p* = 0.58	*r*_s_ = −0.02, *p* = 0.94
Δ Peak plasma nitrate (NO_3_^−^): NO_3_^−^	*r*_s_ = 0.24, *p* = 0.31	***r***_**s**_ = −0.47, *p* = 0.04*	*r*_s_ = −0.06, *p* = 0.79
Δ Peak plasma acetate: INU + NO_3_^−^	*r* = 0.20, *p* = 0.39	*r* = 0.13, *p* = 0.57	*r* = 0.20, *p* = 0.41
Δ Peak plasma acetate: INU	*r* = −0.21, *p* = 0.37	*r* = −0.33, *p* = 0.15	*r* = −0.30, *p* = 0.20

Δ changes in systolic blood pressure (ΔSBP), diastolic blood pressure (ΔDBP), and mean arterial pressure (ΔMAP). Δ changes in peak nitrite, peak nitrate, and peak acetate concentrations in plasma. Abbreviations: INU, inulin; NO_3_^−^, nitrate; NO_2_^−^, nitrite. “*r*_s_” indicates Spearman’s rank correlation coefficient; “*r*” indicates Pearson’s correlation coefficient. *p < 0.05, **p < 0.01.

The author apologises for these errors and wishes to correct them through this notice.
