# Supplementary material for: The effects of inorganic nitrate and inulin co-ingestion on circulating metabolites and blood pressure in young adults: a pilot double-blind randomised crossover trial
Source: Gut Microbiome (Camb). 2025 Jun 26;6:e11. doi: 10.1017/gmb.2025.10008 (PMC12277101; doi:10.1017/gmb.2025.10008)
Supplement: Virgili et al. supplementary material [file S263228972510008Xsup001.zip › Acute-Inorganic-Nitrate-Inulin-Coingestion-Metabolites-BP-Supplementary-GM.docx]

The effects of inorganic nitrate and inulin co-ingestion on circulating metabolites and blood pressure in young adults: A pilot double-blind randomised crossover trial

Jessica Virgili^1^, Gwenaelle Le Gall^2^, Anni Vanhatalo^1^, Bert Bond^1^, David Vauzour^2^, and Luciana Torquati^1^

^1^University of Exeter Medical School, Faculty of Health and Life Sciences, Department of Public Health and Sport Sciences, University of Exeter, St Lukes Campus, Heavitree Road, Exeter, EX1 2LU, UK.

^2^Norwich Medical School, Faculty of Medicine and Health Sciences, University of East Anglia, Norwich, NR4 7TJ, UK

Correspondence:

[l.torquati@exeter.ac.uk](mailto:l.torquati@exeter.ac.uk)

**Keywords:** Fermentable fibre; Nitrate; Gut microbiome; Acetate; Vascular health

**Materials and Methods**

Study Population

Twenty healthy, normotensive, non-smoker participants aged between 18-45 years, with a body mass index (BMI) between 25 and 35 kg/m^2^ were recruited between April and September 2022. Prior to participation, the participants were given a comprehensive explanation and an information sheet detailing the potential risks associated with the study. The study protocol adhered to the core principles of the ICH-GCP and the Helsinki Declaration and was approved by the Public Health and Sport Sciences Ethics Committee (Ethics Committee of the University of Exeter (approval ID: 22-02-02 A-01). All participants provided written informed consent after satisfying inclusion/exclusion criteria, as shown below.

Inclusion criteria:

1. Adults between 18 and 45 years of age, free from cardiometabolic diseases
2. Body mass index (BMI) between 18 and 25 kg/m^2^ or waist-to-hip ratio of 0.85 or less for women and 0.9 or less for men
3. Controlled blood pressure (BP), defined as systolic blood pressure (SBP) of ≤ 120 mmHg and a diastolic blood pressure (DBP) of ≤ 80 mmHg
4. The participant is able to understand and comply with the protocol requirements, instructions and protocol-stated restrictions

Exclusion criteria:

1. Use of antibiotics for three months prior to the study or during the study (Yuan et al., 2023)
2. Any food allergies and/or intolerances
3. Being on a weight loss intervention or following any restrictive dietary practices (vegan, FODMAP, etc.)
4. History of chronic gastrointestinal conditions (e.g., irritable bowel syndrome, inflammatory bowel diseases, coeliac disease, constipation)
5. Pre-existing medical conditions, including hypertension, diabetes, or dental conditions (gingivitis) or any cardiovascular, endocrine, and metabolic disease requiring treatment
6. Use of any medication that influences gut transit or intestinal microbiota (e.g., proton pump inhibitors, metformin, and laxatives) and prebiotics/probiotics for at least one month prior to the study or during the study
7. Use of beta-adrenergic blocking agents, lipid-lowering drugs, and hypertensive medications
8. Pregnant or lactating female participants
9. Regular use of an antibacterial mouthwash or tongue scrapes (McDonagh et al., 2018)
10. Current or recent consumption of NO_3_^−^, L-arginine or L-citrulline supplements.
11. Smoking

Randomisation and blinding

A researcher not involved in participant recruitment carried out the randomisation process using an online tool (www.randomizer.org), generating consecutive sets of numbers ranging from one to three that corresponded with specific interventions. Each participant was sequentially allocated one of six randomisation sequences based on eligibility following the screening visit. The supplementations order remained blinded until data and statistical analysis at the end of the study.

Plasma short-chain fatty acids analysis:

Reagent and chemicals: All aqueous solutions were prepared using purified water at a Milli-Q grade. Acetic acid, propionic acid, butyric acid, isobutyric acid, 2-methylbutyric acid, valeric acid, isovaleric acid, caproic acid, isocaproic acid, acetic-d_3_ (ac-d3), propionic acid-d_2_ (pro-d2) and 2-isobutoxyacetic acid were purchased from Sigma-Aldrich (UK). The solutions were prepared in methanol and stored at −20°C. For derivatisation 3-Nitrophenylhydrazine hydrochloride (3-NPH), 1-Ethyl-3-(3-dimethy-laminopropyl) carbodiimide hydrochloride (EDC) and pyridine were purchased by Sigma-Aldrich (UK).

Sample preparation: 40 µL serum was diluted with 500 µL ice-cold methanol and incubated on dry ice for 15 minutes. The samples were centrifuged (14800 rpm, 5 minutes) and supernatants were filtered using 0.45 µM PTFE filters. The filtered extracts were evaporated until dry using a Savant™ SpeedVac™ High-Capacity Concentrator (Cat. SC210A-230) and were reconstituted with 40 µL of HPLC methanol. 20 µL of reconstituted sample was mixed with 20 µL of internal standard mix (20ppm acetic acid-d3, propionic acid-d2, and iso-butoxy acetic acid). For derivatisation, 10 µL of 3-NPH (50 mM 3-NPH solution) and 10 µL of EDC (50 mM EDC solution) made in 7% pyridine (v/v methanol) were added to the reconstituted extracts and incubated at 37°C for 30 minutes in a Standard Incubator (Model B 28, Binder, Tuttlingen, Germany). The derivatisation reaction was quenched by adding 20 µL of 0.1% formic acid. All the derivatised samples were transferred to the autosampler vials containing 150 µL inserts and run on the LC-MS/MS system. Stock solutions of each metabolite were prepared in methanol (1mg/mL) and stored at -80°C. Calibration standards were prepared by pooling all relevant analytes for each method at five different concentrations by preparing a serial dilution of a solution containing acetic acid at 30 µg/mL, propionic acid at 2.5 µg/mL and the other six SCFAs at 1.25 µg/mL and adding the respective internal standards at 50 µg/mL. Calibration standards were run at the beginning, middle and end of each analytical queue. The analyte: internal standard response ratio was used to create calibration curves and quantify each metabolite.

LC-MS/MS Condition: metabolite quantification was performed using liquid chromatography-tandem mass spectrometry (LC-MS/MS) comprising of Waters Acquity UPLC system and Xevo TQ-S Cronos mass spectrometer controlled by MassLynx 4.1 software. For the detection of SCFAs, the electrospray ionisation (ESI) operated in negative mode and chromatographic separations were performed with a Kinetex® 2.6 µM XB-C18 (50 x 2.1 m, Phenomenex Inc.). Eluent A (0.1% formic acid, water) and eluent B (0.1% formic acid, methanol) ran at a constant rate of 0.5 mL/minute. The gradient began at 10% B and was held for 1 minute before a linear increase to 23% B occurred at 4.5 minutes, then 50% B at 6 minutes following by 100% B at 7 minutes. This was held for 0.5 minutes before a linear decrease in gradient back to 10% B occurred between 7.5 and 12 minutes. This method was adapted from Dei Cas et al. (2020).

Chromatogram peak analysis was performed by the accompanying Waters® TargetLynx ™ application manager and all further data analysis and calibration curve constructions were completed in Microsoft Excel (2019 version).

Method Performance: excellent linear response range was ensured for each calibration curve with correlation coefficients (r^2^) 0.99 or higher for all calibration curves generated. An Agilent high performance autosampler with an injection program was used to minimise carry-over effects between samples. Samples were run in a random order and 20% of the whole set was re-run as a quality control for the method repeatability. No signal was also detected in blank samples run amongst the serum and calibration injections, or in blanks run after the highest calibration standard, indicating that there was little to no carry-over occurring.

Statistical Analysis

This study aimed to investigate the effects of combining nitrate with inulin on NO biomarkers, such as plasma nitrate and nitrite, compared with individual supplements by measuring plasma nitrate and nitrite concentrations from baseline to 360 minutes post-consumption. Additionally, this study assessed the impact of the supplements on plasma SCFAs (acetate, butyrate, and propionate) concentrations. This study further examined the effects of the supplements on systolic (SBP), diastolic (DBP), and mean arterial blood pressure (MAP) variables over the same period. The relationship between individual peak changes in NO metabolites and SCFA acetate and corresponding BP changes was explored. The peak concentrations for each supplementation were identified as the highest measured values at any timepoint for each individual.

Data were analysed using SPSS (IBM SPSS Statistics, Version 29). Graphical representations were created using GraphPad Prism software (GraphPad Software V 10.1.1; San Diego, CA, USA). Changes over time (0-360 minutes) in plasma nitrite, nitrate, and SCFAs, condition (15 g INU + NO_3_^-^, NO_3_^-^, and INU), and time x condition effects were analysed using a two-way repeated measures ANOVA with time and condition used as within-subject factors. In case of a significant interaction, a post hoc test with Bonferroni correction was performed to compare treatment effects at specific time points. When the interaction effects were not significant, the main effects of time and condition were analysed separately. The total and incremental area under the curve (tAUC and iAUC) were determined by applying the trapezium rule and were calculated using R Statistical Software (R version 4.3.2). tAUC was used to evaluate the overall impact of conditions on the concentrations of plasma nitrate and nitrite and SCFAs. In contrast, iAUC was used to show how the conditions specifically enhanced these metabolites, which is distinct from the general trend in total concentration. Differences in tAUC and iAUC after treatment were compared using a one-way repeated-measures ANOVA. Where significant differences were found, a Bonferroni post-hoc test was performed. ANOVA unstandardised residuals were tested for normality using the Shapiro-Wilk test. Sphericity was checked using Mauchly's test, and multivariate models were applied if the assumptions were unmet. If the data were not normally distributed and sphericity was violated, the Friedman test was used as a non-parametric alternative (Blanca et al., 2023). Correlation analyses, using Spearman’s rank-order and Pearson’s methods depending on normality, were used to investigate the relationships between individual peak changes in nitrite, nitrate, and acetate and the corresponding BP variables timepoints. Correlation strengths were inferred using thresholds of 0.2 for weak, 0.5 for moderate, and 0.8 for strong correlations (Mukaka, 2012). All data are expressed as the mean ± SD, unless otherwise stated. In all cases, statistical significance was set at *p* ≤ 0.05.

**Table S1**: Nutritional content of the three conditions.

| Nutrient | INU + NO_3_^-^ | NO_3_^-^ | INU |
| --- | --- | --- | --- |
| Energy, kcal | 22.5 | 0 | 22.5 |
| Carbohydrates, g | 1.2 | 0 | 1.2 |
| Dietary Fibre, g | 13.5 | 0 | 13.5 |
| Protein, g | 0 | 0 | 0 |
| Fats, g | 0 | 0 | 0 |
| Potassium, mg | 154.8 | 154.8 | 0 |
| Nitrate, mg | 400 | 400 | 0 |

**Abbreviations:** INU = Inulin; NO_3_^-^ = nitrate. INU + NO_3_^-^ treatment included 15 g chicory root Orafti®, 0.4 g potassium nitrate, 300 mL sterile water, 20 mL cordial (sugar-free); NO_3_^-^ treatment included 400 mg potassium nitrate, 300 mL sterile water, 20 mL cordial (sugar-free); INU treatment included 15 g chicory root Orafti®, 300 mL sterile water, 20 mL cordial (sugar-free).

**Table S2**: Nutritional content of the standard lunch provided during each testing visit.

| Nutrient |  |
| --- | --- |
| Energy, kcal | 367 |
| Carbohydrates, g | 44 |
| Dietary fibre, g | 2.2 |
| Starch, g | 41 |
| Sugar, g | 3 |
| Protein, g | 14.1 |
| Fat, g | 15.1 |
| Saturated fat, g | 7.5 |
| Sodium, mg | 558 |
| Chloride, mg | 845 |
| Calcium, mg | 163 |

Nutritional content values for the standard lunch are based on a Nutritics report (Nutritics, 2019). Lunch Ingredients included 94 g white bread, 22 g lactose-free cheddar cheese, and 7 g spreadable plant-based butter.

**Figure S1.** Plasma nitrite and nitrate following INU+NO_3_^-^ (pink), NO_3_^-^ (green), and INU (orange) conditions. A) plasma nitrite (nM) over 360 minutes; B) plasma nitrite tAUC from baseline to 360 minutes; C) plasma nitrate (µM) over 360 minutes; D) plasma nitrate tAUC from baseline to 360 minutes. All results are expressed as means ± SD (*n* = 20). ns p > 0.05, ***p < 0.001. Abbreviations: tAUC, total area under the curve; INU, inulin; NO_3_^-^, nitrate; min, minutes.

**Figure S2.** Plasma acetate, propionate and butyrate following INU+NO_3_^-^ (pink), NO_3_^-^ (green), and INU (orange) conditions. A) plasma acetate (µM) over 360 minutes; B) plasma acetate tAUC from baseline to 360 minutes; C) plasma propionate (µM) over 360 minutes; D) plasma propionate tAUC from baseline to 360 minutes; E) plasma butyrate (µM) over 360 minutes; F) plasma butyrate tAUC from baseline to 360 minutes. All results are expressed as means ± SD (n = 20). tAUC = total area under the curve. ns p > 0.05, **p = 0.001, *p < 0.01. Abbreviations: tAUC, total area under the curve; INU, inulin; NO_3_^-^, nitrate; min, minutes.

**Supplementary References**

Blanca, M. J., Arnau, J., García-Castro, F. J., Alarcón, R., & Bono, R. (2023). Non-normal Data in Repeated Measures ANOVA: Impact on Type I Error and Power. *Psicothema*, *35*(1), 21-29. <https://doi.org/10.7334/psicothema2022.292>

Dei Cas, M., Paroni, R., Saccardo, A., Casagni, E., Arnoldi, S., Gambaro, V., Saresella, M., Mario, C., La Rosa, F., Marventano, I., Piancone, F., & Roda, G. (2020). A straightforward LC-MS/MS analysis to study serum profile of short and medium chain fatty acids. *J Chromatogr B Analyt Technol Biomed Life Sci*, *1154*, 121982. <https://doi.org/10.1016/j.jchromb.2020.121982>

McDonagh, S. T. J., Wylie, L. J., Webster, J. M. A., Vanhatalo, A., & Jones, A. M. (2018). Influence of dietary nitrate food forms on nitrate metabolism and blood pressure in healthy normotensive adults. *Nitric Oxide*, *72*, 66-74. <https://doi.org/10.1016/j.niox.2017.12.001>

Mukaka, M. M. (2012). Statistics corner: A guide to appropriate use of correlation coefficient in medical research. *Malawi Med J*, *24*(3), 69-71. <https://www.ncbi.nlm.nih.gov/pmc/articles/PMC3576830/pdf/MMJ2403-0069.pdf>

Yuan, X., Zhou, F., Wang, H., Xu, X., Xu, S., Zhang, C., Zhang, Y., Lu, M., Zhang, Y., Zhou, M., Li, H., Zhang, X., Zhang, T., & Song, J. (2023). Systemic antibiotics increase microbiota pathogenicity and oral bone loss. *International Journal of Oral Science*, *15*(1), 4. <https://doi.org/10.1038/s41368-022-00212-1>
