# Supplementary figures and images for: The effects of inorganic nitrate and inulin co-ingestion on circulating metabolites and blood pressure in young adults: a pilot double-blind randomised crossover trial
Source: Gut Microbiome (Camb). 2025 Jun 26;6:e11. doi: 10.1017/gmb.2025.10008 (PMC12277101; doi:10.1017/gmb.2025.10008)

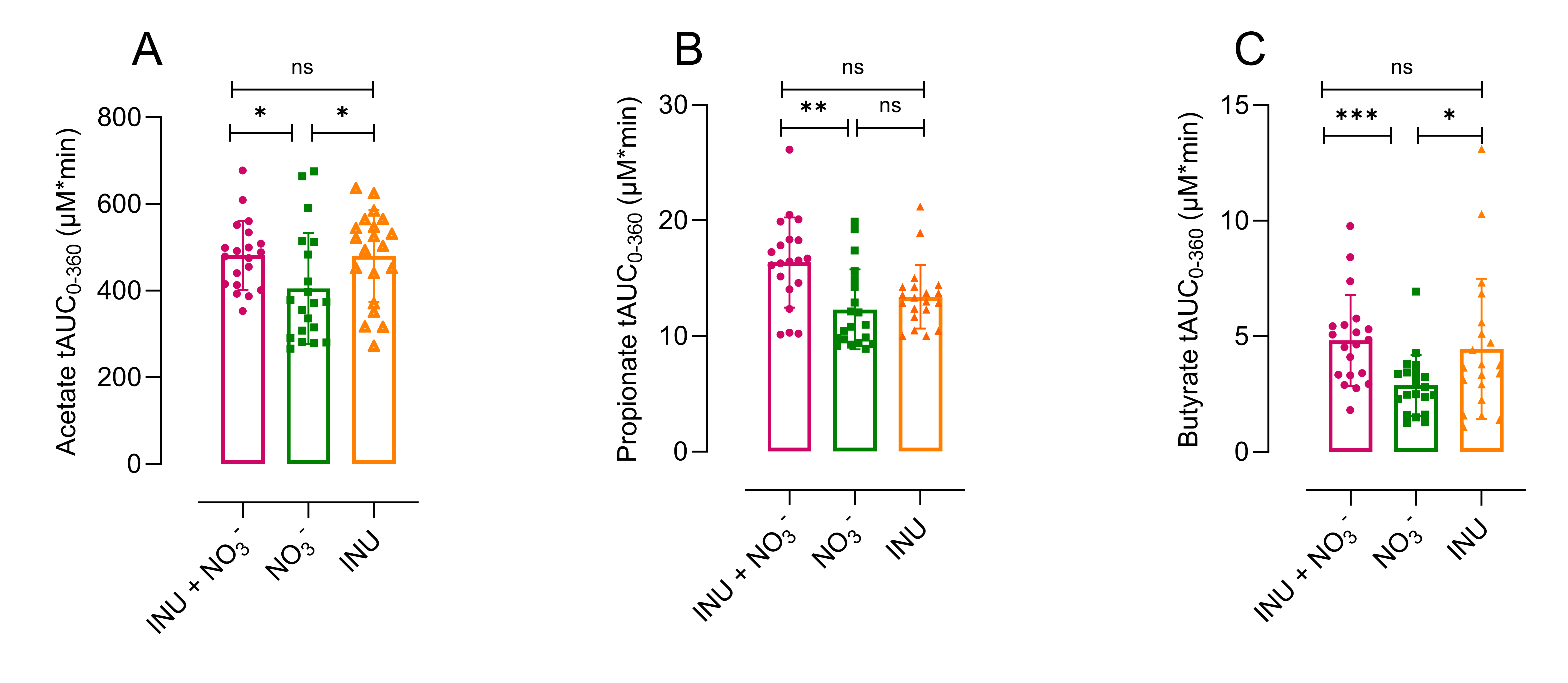

Supplement: Virgili et al. supplementary material [file S263228972510008Xsup001.zip › [Virgili]_Figure S2.600DPI.tif]

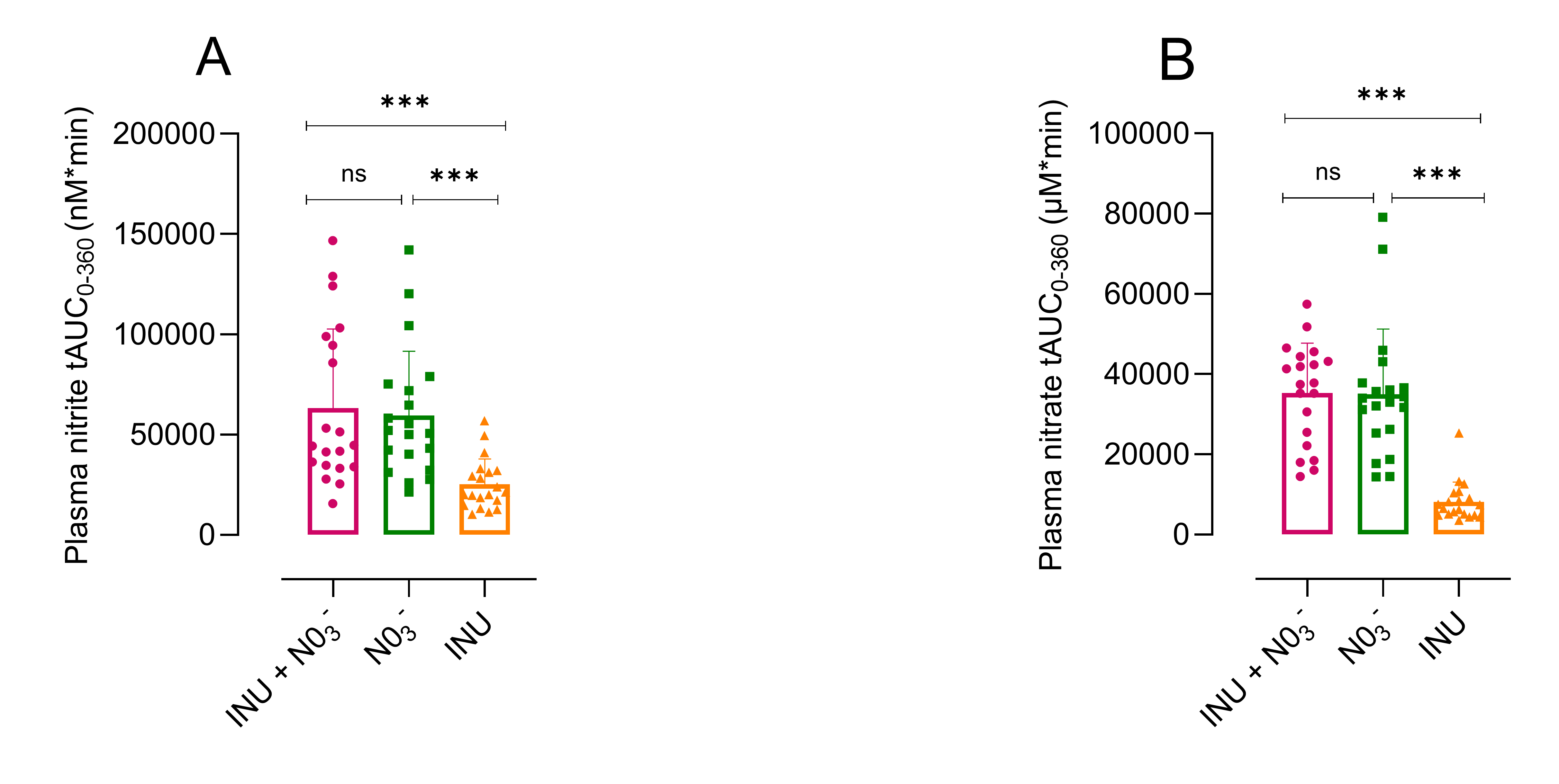

Supplement: Virgili et al. supplementary material [file S263228972510008Xsup001.zip › [Virgili]_Figure S1.600DPI.tif]
